# Development of a Versatile, Low-Cost Electrochemical System to Study Biofilm Redox Activity at the Micron Scale

**DOI:** 10.1128/aem.00434-22

**Published:** 2022-06-27

**Authors:** Alexander D. Klementiev, Marvin Whiteley

**Affiliations:** a School of Biological Sciences, Center for Microbial Dynamics and Infection, Georgia Institute of Technology, Atlanta, Georgia, USA; b Emory-Children’s Cystic Fibrosis Center, Atlanta, Georgia, USA; Novo Nordisk Foundation Center for Biosustainability

**Keywords:** electrochemistry, *Streptococcus*, *Aggregatibacter*, biofilm, redox, topography, *Pseudomonas aeruginosa*

## Abstract

Spatially resolving chemical landscapes surrounding microbial communities can provide insight into chemical interactions that dictate cellular physiology. Electrochemical techniques provide an attractive option for studying these interactions due to their robustness and high sensitivity. Unfortunately, commercial electrochemical platforms that are capable of measuring chemical activity on the micron scale are often expensive and do not easily perform multiple scanning techniques. Here, we report development of an inexpensive electrochemical system that features a combined micromanipulator and potentiostat component capable of scanning surfaces while measuring molecular concentrations or redox profiles. We validate this experimental platform for biological use with a two-species biofilm model composed of the oral bacterial pathogen Aggregatibacter actinomycetemcomitans and the oral commensal Streptococcus gordonii. We measure consumption of H_2_O_2_ by A. actinomycetemcomitans biofilms temporally and spatially, providing new insights into how A. actinomycetemcomitans responds to this S. gordonii-produced metabolite. We advance our platform to spatially measure redox activity above biofilms. Our analysis supports that redox activity surrounding biofilms is species specific, and the region immediately above an S. gordonii biofilm is highly oxidized compared to that above an A. actinomycetemcomitans biofilm. This work provides description and validation of a versatile, quantitative framework for studying bacterial redox-mediated physiology in an integrated and easily adaptable experimental platform.

**IMPORTANCE** Scanning electrochemical probe microscopy methods can provide information of the chemical environment along a spatial surface with micron-scale resolution. These methods often require expensive instruments that perform optimized and highly sensitive niche techniques. Here, we describe a novel system that combines a micromanipulator that scans micron-sized electrodes across the surface of bacterial biofilms and a potentiostat, which performs various electrochemical techniques. This platform allows for spatial measurement of chemical gradients above live bacteria in real time, and as proof of concept, we utilize this setup to map H_2_O_2_ detoxification above an oral pathogen biofilm. We increased the versatility of this platform further by mapping redox potentials of biofilms in real time on the micron scale. Together, this system provides a technical framework for studying chemical interactions among microbes.

## INTRODUCTION

Cellular physiology is dictated largely by the surrounding chemical environment and, as a result, guides certain behavior. This is particularly true of bacteria, which often form surface-associated communities called biofilms that have distinct physiological responses to chemical signals and cues produced by neighboring microbes (1–3). These signals and cues vary from small by-products (1) to more complex polymeric or even toxic molecules intended to attack the host or nearby microbes (4, 5). Together, the chemical properties of the surroundings are critical to modulating the function of virtually all microbial communities, affecting how they develop, change, and cause disease.

Microbial community interactions occur at the micron scale, requiring specialized techniques to spatially map and quantitate chemical information (6, 7). Optical (e.g., methylene blue) (8, 9), chemical (e.g., Winkler titration) (10), and electrochemical (e.g., Clark electrode) (11–15) methods can reveal the local chemistry *in situ*; however, electrochemical studies are ideal for our study, particularly scanning electrochemical microscopy (SECM) (16–19). SECM is both a technique and an instrument that positions an ultramicroelectrode (UME) with a micron-sized tip a known distance away from a surface and then spatially maps the concentrations of molecules (20, 21). SECM has quantified small molecules surrounding microbial biofilms on the micron scale and, more recently, individual cells (18, 22–24). However, scanning electrochemistry experimental platforms often require specialized equipment, which is extremely expensive and, while highly sensitive, technically limited. For example, SECM is a powerful tool that measures redox-active molecules in solution and performs in a mode that measures electrical currents while scanning (13, 18, 24). Users must purchase additional pieces of equipment (25) to perform more complex experiments while continuously mapping chemical landscapes, and this often deters researchers not familiar with electrochemistry. The technical limitations of SECM led the field of electrochemistry to pursue other experimental systems that offer greater flexibility and incorporate new techniques (7).

Here, we develop and validate a new, low-cost electrochemical system and accompanying analysis suite that capture a breath of electrochemical techniques while spatially measuring chemical information on the micron scale. We present a roadmap for building this electrochemical toolkit using new and inexpensive equipment that requires minimal out-of-pocket costs with a means for in-lab fabrication of UMEs at <$6 and an arsenal of equipment for fabricating these UMEs at <$10. This provides the average laboratory the ability to perform an array of electrochemical experiments at low costs.

We validated this electrochemical platform for biological applications using a two-species bacterial biofilm model composed of Streptococcus gordonii and Aggregatibacter actinomycetemcomitans. S. gordonii is a human commensal bacterium found most commonly on the surface of teeth, where it serves as an early colonizer (26). S. gordonii is capable of outcompeting neighboring bacteria by lowering the pH (25) through l-lactic acid production (1) and reactive oxygen species damage by producing hydrogen peroxide (H_2_O_2_) (27). Over time, a sticky matrix surrounds the bacteria and forms a pellicle structure that allows for other bacteria to colonize the tooth (28). Eventually, complex communities form underneath the gum line in the gingival crevice and, if left unchecked, progress to gingivitis and periodontitis, gum inflammation and gum disease, respectively (29). Characterized by bleeding and swelling of the gums, periodontitis can eventually lead to irreversible receding of gums and tooth loss (30). A. actinomycetemcomitans is a key oral plaque pathogen that can be found in aggressive periodontal disease (31). A. actinomycetemcomitans and S. gordonii form complex chemical networks that synergistically benefit both bacteria (1, 2). S. gordonii-produced l-lactic acid serves as a carbon source for A. actinomycetemcomitans (1), and A. actinomycetemcomitans-produced catalase mitigates the harmful effects of H_2_O_2_ produced by S. gordonii (2, 32).

While past electrochemical work has quantified chemical gradients surrounding A. actinomycetemcomitans and S. gordonii biofilms (22, 33), certain aspects of bacterial physiology of both organisms, including the H_2_O_2_ detoxification rate by A. actinomycetemcomitans, are still not known. A. actinomycetemcomitans spatially organizes around H_2_O_2_-producing bacteria, and past work assessed the bacterial organization on the micron scale (2) but did not measure H_2_O_2_ gradients leading to these spatial organizations. We illustrate the ease with which our electrochemical platform can be set up to both map H_2_O_2_ gradients and acquire consumption rates. The platform was then modified to measure redox activity above bacterial biofilms without requiring additional hardware modifications. While redox potential measurements have been observed above biofilms (34–37), we advanced the field by developing SECM-based methods for surface area mapping on the micron scale.

## RESULTS AND DISCUSSION

### UME fabrication.

UMEs can be fabricated using various materials, especially at the sensing tip, which affords them the flexibility to quantify many molecules known to mediate bacterial interactions. In order to study chemical interactions occurring among bacteria, we designed and built in-house equipment for UME fabrication. This includes a UME-sealing apparatus, a polishing wheel, and a modified microscope (see Fig. S1 in the supplemental material). Using the equipment, we fabricated UMEs for all experiments presented in this work.

### Developing a novel experimental platform for electrochemically scanning biofilm surfaces.

**FIG 1:**
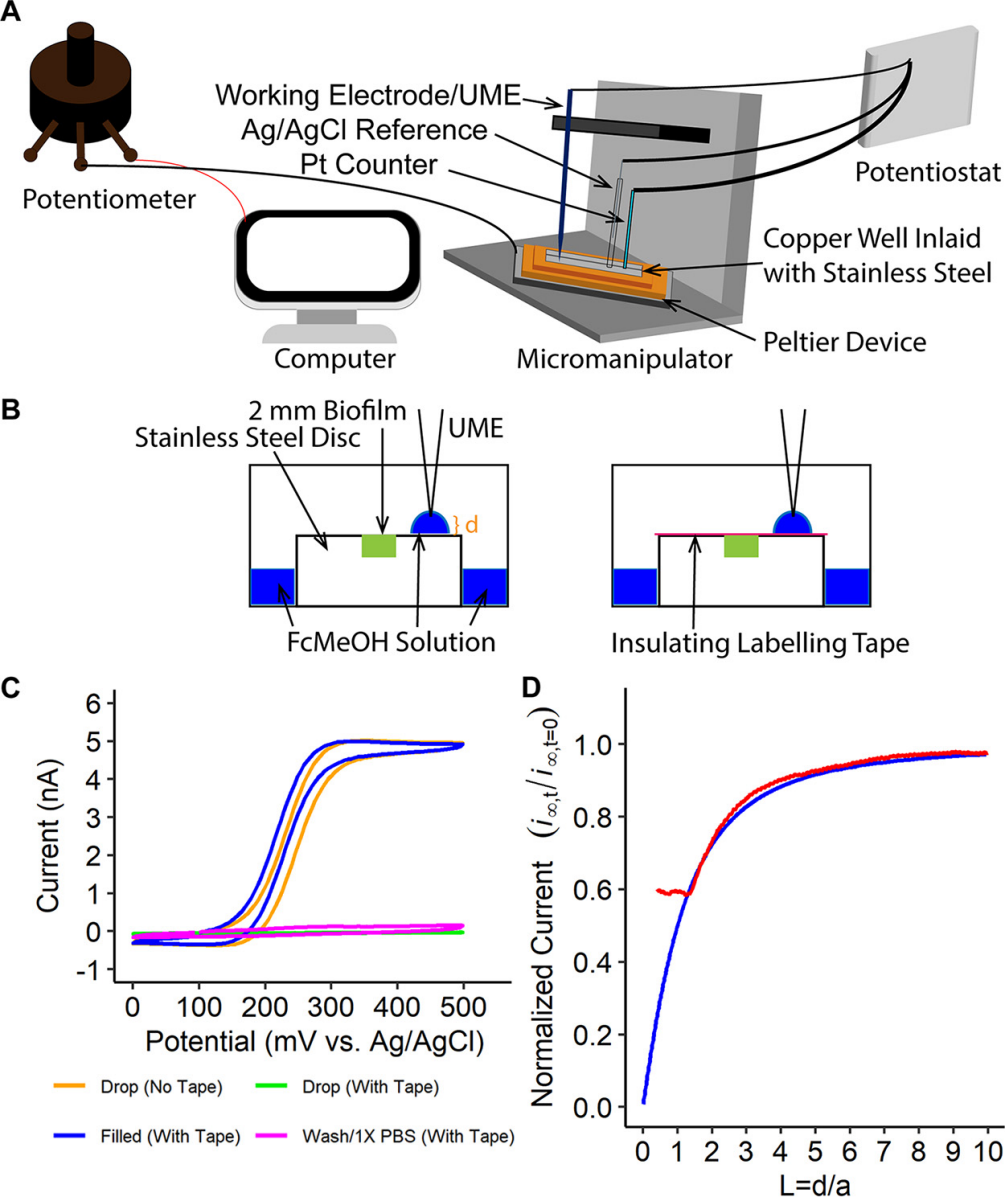
Schematic of the electrochemical system. (A) All electrochemical experiments used a three-electrode setup including a reference, counter, and working electrode. The electrical potential at the UME (also referred to as the working electrode) is set against a known reference electrode potential, and the current is then measured between the working and counter electrode. (B) Schematic of approaching surfaces using the drop method. The approach method had the stainless-steel disc attached to the bottom of the electrochemical cell with double-sided tape (not shown). A total of 2 mL of ~1.5 mM FcMeOH in 1× PBS surrounded the disc. A ~20 to 30 μL drop of the same solution was held suspended on the UME tip as it approached the surface, at which time it formed a drop on the surface of the stainless-steel disc away from the biofilm. The distance (d) approached is shown in orange. On the right, the same image appears with the insulating labeling tape which was used to confirm conduction along the stainless-steel disc. (C) Control experiments verifying the electrochemical setup. A representative cyclic voltammogram using a 25 μm gold UME. All replicates are shown in Fig. S3. Controls include cyclic voltammetry performed in the drop with no tape, in the drop with tape, in bulk solution with tape present on the disc, and when the setup is washed 3× with 1× PBS and then filled with 1× PBS with tape as represented by the orange, green, blue, and magenta cyclic voltammograms, respectively. (D) Representative approach curve to stainless-steel disc and expanded upon in Fig. S4. L, normalized distance where d = actual distance and a = radius of the UME. The blue line represents the expression to which the data (red) were fitted. Here, *i_∞_* represents the current measured away from the surface. Of note, the left trail of the red line was left to indicate the stability of the current after approaching the surface.

Various electrochemical methods are capable of measuring chemical landscapes produced by microbes on the micron scale (7, 20, 38). For our platform, we drew inspiration from SECM with the goal of developing a highly versatile system for measuring chemical environments. This setup incorporates a potentiostat, a basic electrochemical instrument that on its own is capable of performing a range of measurements. Similar to SECM, we combined the potentiostat with a micromanipulator capable of spatially positioning UMEs for electrochemical measurements by the potentiostat (Fig. 1A, Fig. S2).

The main advantage of using SECM as the basis for our design rests on the premise of being able to position the UME specific distances from a surface of interest based on feedback from an electrochemical mediator, commonly a ferrocene-based molecule (39). As the UME approaches the surface, the decreasing gap between the UME and the surface hinders diffusion to the UME surface. While ferrocene species, including ferrocene methanol (FcMeOH), are nontoxic to some cells, the presence of these mediators means that other molecules, such as H_2_O_2_, cannot be measured accurately in their presence due to overlapping current signals (40). Thus, there is a need to develop alternative methods for approaching the surface of biofilms.

We sought to address this need by developing an experimental platform in which biofilms are grown in a location separate from where the surface used for the FcMeOH approach curve is performed. Importantly, the surface of the biofilm and the approach curve surface are level. Thus, the UME can approach the surface using FcMeOH before relocation to the biofilm, resulting in the UME at a known distance above the biofilm. The experimental setup includes a stainless-steel disc that sits at the bottom of a copper chamber inlaid with stainless steel (to prevent copper leaking and potentially disturbing bacterial cells, while also allowing for thermal conductivity for heating) (Fig. 1A). The stainless-steel disc contained a 2-mm-diameter well where the biofilm was grown (Fig. 1B). Biofilms were formed by embedding bacteria in melted 1.5% Noble agar, and this was added to the 2-mm well and allowed to solidify.

We initially performed control experiments using 1.5% Noble agar with no bacterial cells in the biofilm well. The UME was lowered adjacent to the biofilm well while balancing an ~20 μL drop of 1.5 mM FcMeOH until the drop contacted the stainless-steel disc surface and spread across the surface but did not touch the biofilm. At the bottom of the chamber, 2 mL of the same FcMeOH solution was added, which was enough to cover the bottom surface and surround the disc but not enough to submerge the disc. This allowed us to treat the stainless-steel disc like a conductor that has charge distributed along the surface. Thus, the FcMeOH drop on top is electrically connected with the FcMeOH pool below (Fig. 1B), which was confirmed by the ability to measure current in the FcMeOH drop (Fig. 1C, Fig. S3).

We next tested whether an approach curve to the stainless-steel surface adjacent to the biofilm could be performed using the FcMeOH drop. First, we compared the current measured in the FcMeOH drop to the current measured when the same UME was submerged completely in solution with FcMeOH. The average current differed by only 2.9% (Table S1), and the distance was not affected by washing the setup (Table S2); thus, the current measured in the drop was stable and of sufficient magnitude to perform an approach curve. As a control, we measured the current in the drop when the surface of the stainless-steel disc was covered with an insulating material; in this case, we used standard laboratory tape. As expected, no current was measured when the tape was present (Fig. 1C, Fig. S3). With these controls completed, we performed an approach curve to the stainless-steel surface using the FcMeOH drop. As the UME approached the surface of the stainless-steel disc, we observed the expected reduction in current due to hindered diffusion of FcMeOH to the UME tip surface (Fig. 1D, Fig. S4). To ensure that the distance from the surface obtained using the FcMeOH drop was similar to the distance measured using the standard approach curve technique, the entire cell was flooded with FcMeOH solution to cover the disc and an approach curve to the stainless-steel disc was performed. The distances were identical; thus, the drop is a suitable method for approaching surfaces accurately and has the added benefit of not exposing growing biofilms to FcMeOH.

### Quantifying H_2_O_2_ above an A. actinomycetemcomitans biofilm.

**FIG 2:**
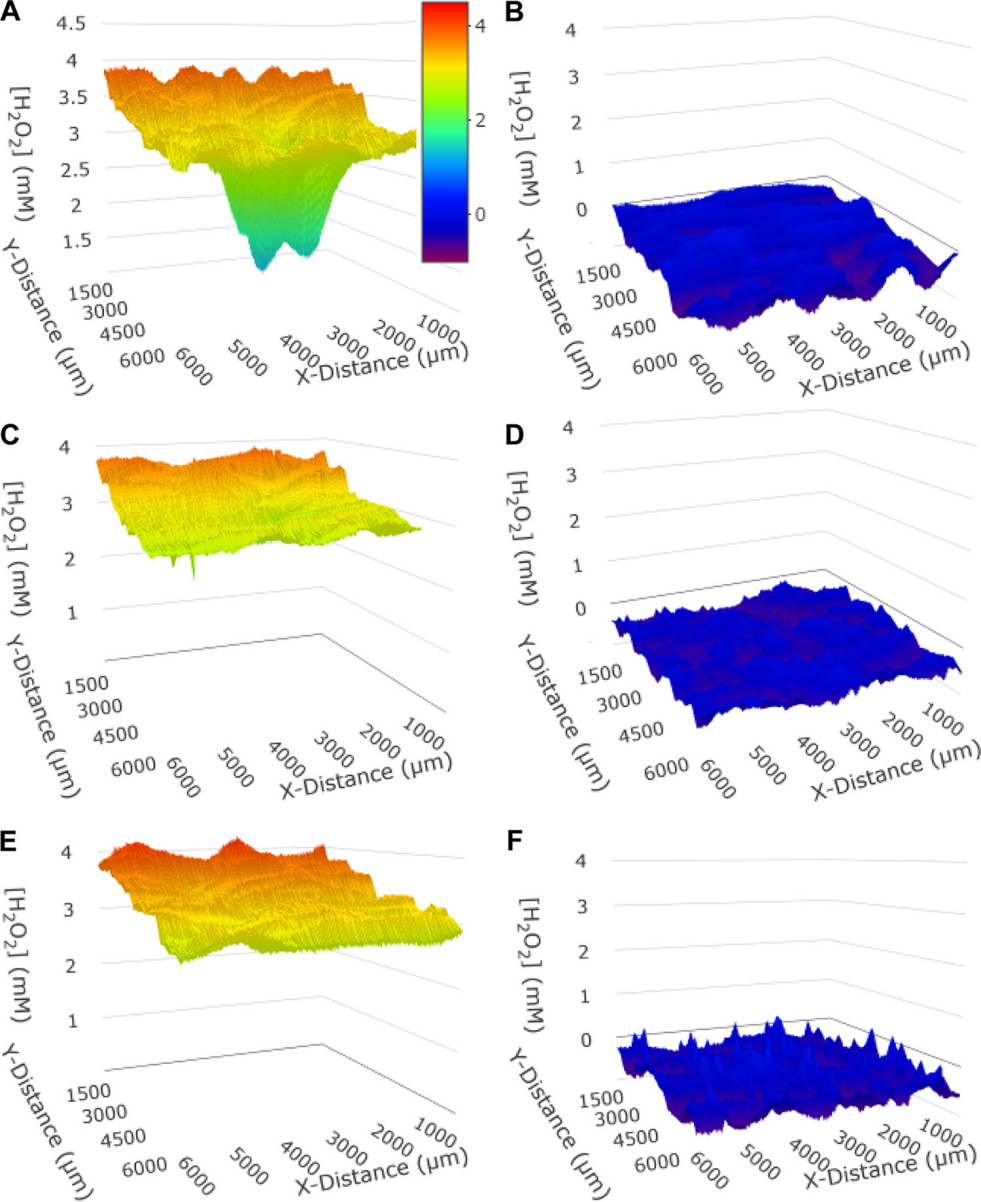
H_2_O_2_ detoxification profiles 100 μm away from a 2 mm A. actinomycetemcomitans biofilm. (A) WT A. actinomycetemcomitans with H_2_O_2_ added showed a decrease in local H_2_O_2_ levels near the biofilm. (B) WT A. actinomycetemcomitans without H_2_O_2_ added showed no measurable H_2_O_2_ above biofilms. (C) The A. actinomycetemcomitans *katA*^−^ mutant with H_2_O_2_ added showed a small decrease in local H_2_O_2_ levels near the biofilm. (D) A. actinomycetemcomitans *katA*^−^ mutant without H_2_O_2_ added showed no measurable H_2_O_2_ above biofilms. (E) Positive control of medium with added H_2_O_2_ and no bacteria. (F) Negative control of media with no H_2_O_2_ and no bacteria. All decreases in signal over time (shown as Y-distance increases) are due to fouling of the UME surface, which is expected for all experiments due to the hour-long scanning time.

Having established a working experimental platform, we next sought to apply our instrumentation to quantify metabolites surrounding bacterial biofilms. Past work used SECM to measure H_2_O_2_ consumption by A. actinomycetemcomitans biofilms (22), and we sought to validate these results using our experimental system. After confirming the surface along which the UME scans were even (Fig. S5), we developed a culture medium that supported bacterial growth and also allowed for long-term measurement of H_2_O_2_ with minimal electrode fouling (which occurs as a result of molecules in solution binding the UME surface and decreasing current) (Fig. S6 and S7) (41). A. actinomycetemcomitans biofilms were then grown in the biofilm well and exposed to 4 mM H_2_O_2_, and the UME quantified H_2_O_2_ levels while scanning 100 μm above the biofilm over the entire surface (Fig. 2, Fig. S8 to S10). Despite expected electrode fouling leading to signal decay, H_2_O_2_ levels decreased by 50% above the center of the biofilm, similar to what was observed in previous experiments with SECM (22). As a control, biofilms were also formed using an A. actinomycetemcomitans strain (*katA*^−^) that does not produce the H_2_O_2_-degrading enzyme catalase. H_2_O_2_ levels above the center of the A. actinomycetemcomitans *katA*^−^ biofilm decreased by only 20% and significantly less than observed for wild-type (WT) A. actinomycetemcomitans, validating previous measurements. These data indicate that our experimental platform can accurately quantify H_2_O_2_ above the surface of an A. actinomycetemcomitans biofilm, obtaining results similar to those acquired via conventional SECM instruments.

**FIG 3:**
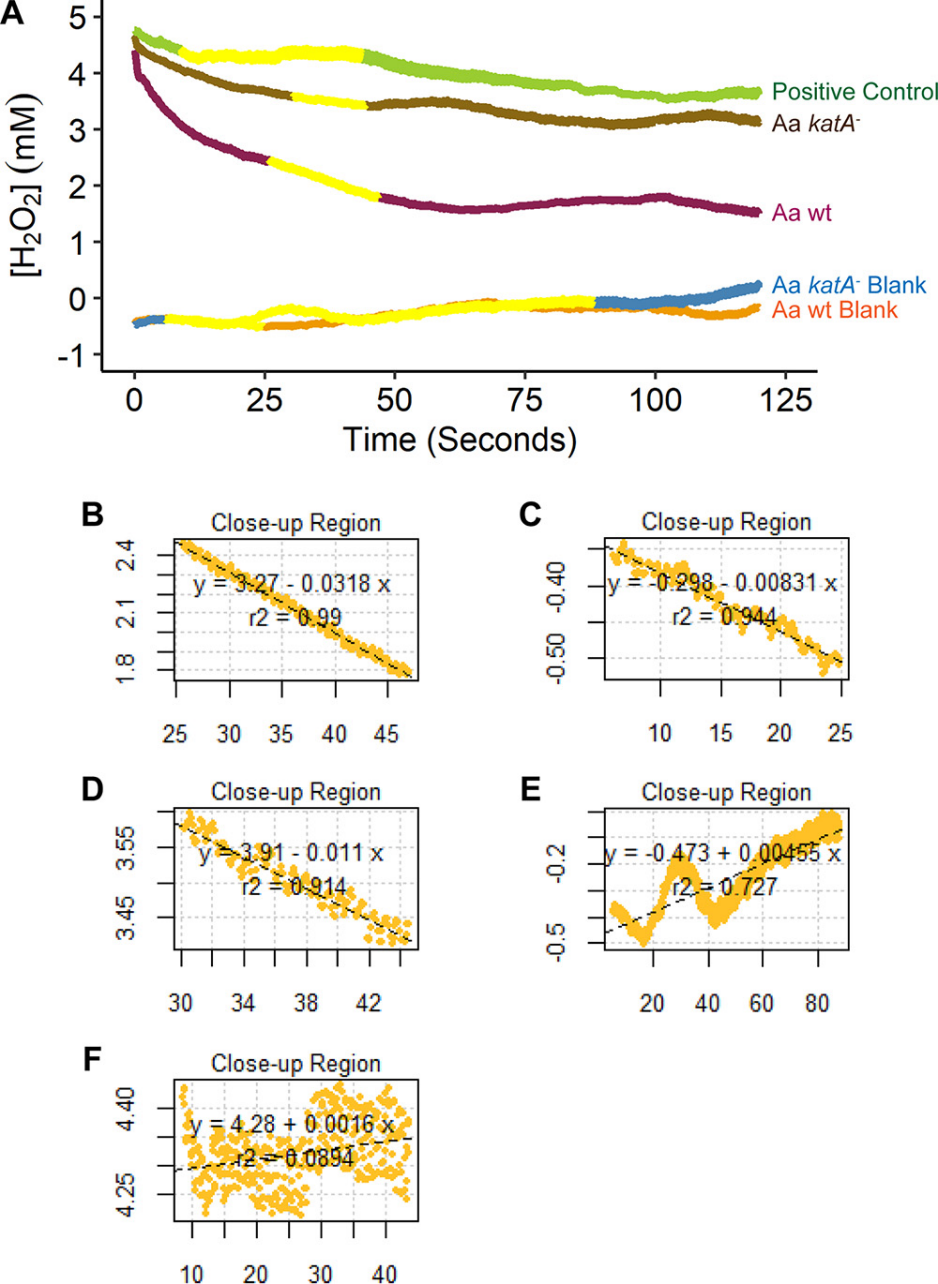
Consumption of exogenous H_2_O_2_ 100 μm above A. actinomycetemcomitans biofilms. (A) H_2_O_2_ consumption by WT A. actinomycetemcomitans and A. actinomycetemcomitans *katA*^−^. Blank conditions contain no H_2_O_2_, and the positive control is H_2_O_2_ with no bacteria. The yellow portion of the curve was used by RespR. (B to F) Calculating the rate of H_2_O_2_ breakdown using RespR for (B) WT A. actinomycetemcomitans with H_2_O_2_ added, (C) WT A. actinomycetemcomitans without H_2_O_2_, (D) A. actinomycetemcomitans *katA*^−^ with H_2_O_2_ added, (E) A. actinomycetemcomitans *katA*^−^ without H_2_O_2_, and (F) positive control of medium with H_2_O_2_ and no bacteria. Here, the slope is equivalent to the best-fit rate in Table S3 (*n* = 3 to 5).

Previous work used simulated models to estimate kinetics of H_2_O_2_ production (22), although the kinetics of consumption have not been examined. To quantify consumption rates, H_2_O_2_ levels were quantified by measuring current with the UME over time above an A. actinomycetemcomitans biofilm (Fig. 3A). A publicly available computational package, RespR, was adapted to quantify H_2_O_2_ consumption rates (42). RespR detects the most linear sections of data along the current curve using novel rolling regression and kernel density estimation techniques to measure a best-fit rate for concentration changes over time (Fig. 3B to F). The use of multiple linear sections is critical, as the initial portions of the curve may not be indicative of H_2_O_2_ consumption, while late regions may be the point when biofilms and natural mass transfer of H_2_O_2_ to the bacterial surface are reaching equilibrium. Best-fit rates for H_2_O_2_ degradation revealed an average rate by A. actinomycetemcomitans (32 μM/s) and a lower rate by A. actinomycetemcomitans *katA*^−^ (11 μM/s) (Table S3). These data reveal that catalase accounts for a majority of the H_2_O_2_-degrading activity of an A. actinomycetemcomitans biofilm and that our system can sensitively measure H_2_O_2_ consumption rates.

### Measuring redox activity above biofilms.

Having demonstrated the utility of our electrochemical system for quantifying H_2_O_2_ levels, we next turned to the myriad of techniques the potentiostat is capable of performing, which include common DC sweep, step, open circuit, and chronopotentiometry experiments. Having measured current, we chose to measure electrical potentials, and while open circuit potential has practical application and is a technique the platform is capable of performing, it is mainly a tool for more advanced methods (43). On the other hand, chronopotentiometry has had a broader range of application from calculating diffusion coefficients of non-redox-active species (44) to measuring real-time changes in biofilm thickness (45).

In general, the redox potential is a quantitative measure of the overall reducing or oxidizing capacity of a system and does not describe a single reaction. On the micro scale, redox potentials dictate metabolism (46) and antibiotic efficacy (47), and on the macro scale, redox potentials can describe nutrient dynamics such as nutrient cycling (48, 49). Past work has demonstrated that bacteria can sense changes in redox potential and react by regulating gene expression and metabolic pathways (50). Thus, measuring redox potentials around a biofilm is critical to understanding their biology.

**FIG 4:**
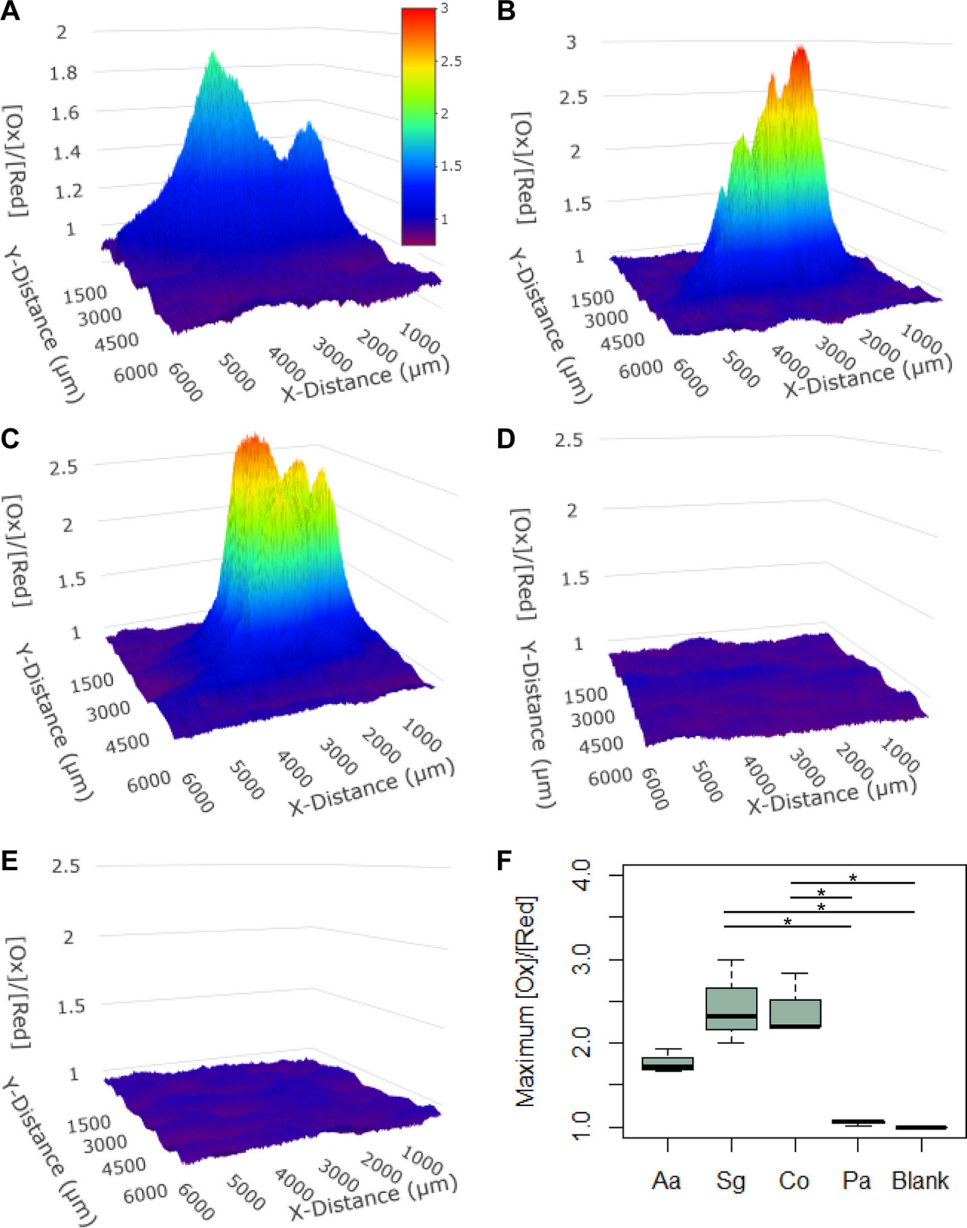
Chronopotentiometry scans 100 μm from the surface of bacterial biofilms. Scanning (A) A. actinomycetemcomitans, (B) S. gordonii, (C) A. actinomycetemcomitans/S. gordonii, (D) P. aeruginosa, and (E) medium control with no bacteria. (F) Maximum differences in redox activity for all biofilms. ANOVA revealed that there are statistically significant differences between conditions (F = 15.3031, *P* = 0.0003). We performed a *post hoc* Turkey HSD test on the data that identified statistically significant groups. *, *P < *.05.

Therefore, we adapted our platform to perform chronopotentiometry to quantify the redox activity surrounding biofilms. We positioned the UME 100 μm above biofilms and set the potentiostat to measure potential changes while applying a current of 0.5 nA. The UME then scanned over the surface of A. actinomycetemcomitans, S. gordonii, and A. actinomycetemcomitans/S. gordonii biofilms. These data revealed differences in redox potential ([Ox]/[Red]) above these biofilms (Fig. 4A to E, Fig. S11 and S12) with both the S. gordonii and A. actinomycetemcomitans/S. gordonii biofilms having levels of oxidized species above the biofilm 2.4-times higher than those of the blank control, while the A. actinomycetemcomitans biofilm was not statistically different from the blank (Fig. 4F, Table S4). We also measured the redox activity above biofilms formed with the bacterium Pseudomonas aeruginosa, which is well known to produce large numbers of reduced molecules (7). Surprisingly, P. aeruginosa biofilms had no detectable differences in redox potential 100 μm above the biofilm surface compared to that of blank controls (Fig. 4F). Past experiments have observed that differences in redox profiles can be expected for different bacterial species (34, 35); therefore, this further supports that bacterial strains may be “typed” based on their ability to influence the redox environment surrounding the biofilm.

Why do S. gordonii-containing biofilms have a high activity of oxidized species above the surface? While the answer is not known, one possible explanation is that the 23-mV change could be due to pH changes above the biofilm as S. gordonii produces large amounts of l-lactic acid. It is known that redox potential increases with a decrease in pH (49) and a 23-mV potential change would correspond to a pH change of 0.39 (51) (Fig. S13A). We tested the role of pH on redox potential by adding a concentration of l-lactic acid to the growth medium that decreased the pH by this amount. Although this pH decrease resulted in a more oxidized ([Ox]/[Red]) environment (Fig. S13B), the redox potential above S. gordonii biofilms was still significantly higher, indicating that other factors are likely important for establishing the highly oxidized environment above these biofilms.

Although S. gordonii required 2 h to change the chemical environment, it was surprising to see no changes in redox activity above P. aeruginosa biofilms. P. aeruginosa is known to manipulate the redox state of its environment by producing phenazine molecules (52), after a sufficient amount of time has allowed for buildup of quorum sensing molecules (53, 54). Perhaps P. aeruginosa requires more time to form these expected steep redox gradients. Future work using this platform may be able to pinpoint the exact time and the rate of onset for redox gradient formation. Furthermore, it is known that the specific molecules not only affect redox state but in the case of P. aeruginosa are also involved in multispecies interactions (55). For example, phenazines can cause reactive oxygen species stress in neighboring organisms (52). Given the dual capability of measuring both redox potential and molecular concentrations, this platform has potential to study complex chemical interactions among microbes on the micron scale.

While past work used potentiometric techniques to study streptococci (25), these measurements required modifications that included an additional potentiostat coupled to an SECM and an optimized pH microprobe (25). Our chronopotentiometry method builds upon our fundamental electrochemical platform and advances the field with newly developed data-processing methods, providing a framework and the initial steps for advancing current industry-standard SECM. Thus, we are able to scan biological surfaces for redox activity with the same ease of measuring H_2_O_2_ concentration profiles over time at the micron scale. Future work can adapt this platform to measure chemical information above more traditional biofilms (such as those grown on filter discs and fed from below) as well as biofilms formed with agar layers on top.

### Conclusions.

Microbiology is a rapidly evolving field that relies on interdisciplinary techniques for understanding microbial physiology. When viewed through the lens of microbiology, electrochemistry provides a straightforward and robust way to measure and detect electroactive molecules and redox potential. While streamlined SECM methods allow for measuring certain aspects of the chemical environment in biological systems, these methods are usually expensive and limited in operable techniques that can be performed with high sensitivity. Here, we present several methodologies for building the necessary components to perform electrochemistry experiments on biological samples at minimal cost. Furthermore, we have shown that our novel electrochemical platform is capable of performing both chronoamperometry and chronopotentiometry techniques while scanning surfaces, and this can be further expanded to include an arsenal of techniques available to potentiostats such as pulse methods. While it is tailored to microbiology studies, we believe that this system can be applicable to a broad range of electrochemical research.

## MATERIALS AND METHODS

### UME fabrication.

UMEs were fabricated as described elsewhere (56). Briefly, a platinum or gold wire (Goodfellow Metals, Cambridge, UK) was sealed in a thick-walled capillary (either 2 mm OD by 0.6 mm ID or 3 mm OD by 0.5 mm ID) using the in-house glass capillary sealer. UME surfaces were polished with the in-house repurposed centrifuge polishing wheel and visualized on a modified microscope to ensure a ratio of radius of glass to radius of sealed metal wire (RG) was ~8 to 10.

### Micromanipulator.

For our instrumentation, we selected the Zaber model, M-LSM050A050A050ARHF-T4A-ENG3187, due to cost, ease of programming, and flexibility of the company to customize equipment and add-ons. The specifications included 3 axis control, 50 mm travel for *xyz* axes, right-handed operation, base mount, X-MCC multiaxis universal motor controller, and an X-JOY3 programmable 3-axis joystick.

### Potentiostat/electrochemical cell.

In our three-electrode system, the potential is measured between the working (UME) and reference electrode (Ag|AgCl|Saturated KCl); the current is measured between the working and counter electrode (platinum wire). The most important factors we considered when selecting the potentiostat included being able to measure current at picoampere ranges, maintaining current stability without significant noise interference, having a reasonable potential window (± 4 V), and being capable of performing multiple techniques (cyclic voltammetry [CV], chronoamperometry [CA], and cyclic step chronoamperometry [CSCA]). For our potentiostat, we selected the WaveNano from Pine Research.

The micromanipulator and potentiostat were placed on an optical breadboard for minimizing electrical noise (Newport Corp.). For all experiments, heat was supplied from a Peltier device with a constant current running through it with the temperature set to 31 ± 2°C via an additional variable resistor to ensure heating without significant evaporation within the electrochemical cell. For all chronoamperometry and chronopotentiometry experiments with biofilms, the working electrode was a 25-μm Pt UME with an RG of ~8 to 10.

### Software setup.

All software, instruments, and machines were run on a 3.2 GHz Intel Core i3-550 processor and 4 GB of RAM desktop PC. All software was current and updated when possible and compatible with the current operating system. The necessary software drivers were updated and installed.

### Proprietary software.

For the micromanipulator, we used Zaber Console for UME positioning and movement. In addition to the software, we also purchased a joystick controller with buttons for programming functions such as disabling axes. For some experiments, it may be necessary to disable certain axes; for example, when scanning in the *z* axis, the other two axes may be disabled to minimize noise. For the potentiostat, we used AfterMath (version 1.5.9885) as provided by Pine Research for all electrochemical experiments.

### Additional software.

The movement of the micromanipulator was programmed using C# (in Visual Studio), in which the scan speed and distance of each axis are first specified and then the number of iterations is specified. The script runs iterations in a snake-like pattern but can be changed to suit the user’s needs. The script is available in the supplemental material.

### Chronopotentiometry baseline correction and data processing.

For chronopotentiometry experiments, it was necessary to do a baseline correction to account for electrode fouling. Over time, it is expected that molecules in solution will stick to the electrode surface and therefore require the instrument to increase the potential to maintain the set current (see Princeton Applied Research Application Note E-4 for more information). To overcome this limitation, we adapted a Python script discussed elsewhere (https://bit.ly/314Tku3) for baseline subtraction using a lambda value of 1e^8^ and a *P* value of 0.0001, which gave a smooth correction. For each individual replicate, we subtracted the minimum value for that replicate from all points to adjust for differences between replicates and then subtracted the average maximum value of all the negative-control blanks to set the threshold as the maximum noise level. These data were then solved for to obtain the [Ox]/[Red] values for each point using equation 1 (below).

(1) $$\frac{E-E^{0}}{-0.0263\text{ V}}=\ln\left(\frac{\left[\mathrm{Red}\right]}{\left[\mathrm{Ox}\right]}\right)$$

Here, *E* is the potential measured, *E*^0^ is the average potential of medium control biofilm scans, and [Red]/[Ox] corresponds to the relative activity of reduced species to that of oxidized species. To obtain the maximum redox activity for all experimental conditions, we took maximum potential values for each replicate, solved for redox activity using equation 1, and then averaged values across replicates. The baseline correction script is available in the supplemental material.

### Processing scanned data and forming images.

Once continuous chronoamperometry or chronopotentiometry data were obtained, we took the data from AfterMath (either time and current or time and potential) and created a spreadsheet in CSV format with the following columns: Time.M (time in minutes from raw data), Time.S (time in seconds of raw data), Time.SA (time in seconds shifted by starting amount), and I or V (raw current or potential for each time point). The zero-time point was manually adjusted (to obtain Time.SA) by inspecting the data and finding the time difference between when the potentiostat started collecting data and when the micromanipulator began movement. For all experiments, the potentiostat was initiated first with the understanding that charging current would be removed as the data corresponding to the start of the micromanipulator a few minutes later are removed. All images were processed using an in-house R script and are available in the supplemental material. Once the data table was imported, the script segmented linear portions and aligned them with micromanipulator movements to produce images. The R script also allows for input of the slope and *y* intercept of a calibration curve that allows for the conversion of current to concentration for the *z* axis. Finally, the script creates a mesh image of the electrochemical information studied.

### Approaching surfaces.

The stainless-steel disc was attached to the bottom of the electrochemical cell using double-sided tape and leveled using a bubble level before each measurement. Surrounding the stainless-steel disc was 2 mL of ~1.5 mM FcMeOH in 1× phosphate-buffered saline (PBS), and an ~20 to 30 μL drop of the same solution was suspended on the tip of the UME. Using our three-electrode setup, the UME tip was lowered to the surface until the drop made contact with the stainless-steel surface and current was measured. As the tip approached the surface, a decrease in current signaled hindered diffusion, and distances were set by fitting the data to an expression (57).

### Calibration curve for H_2_O_2_.

A standard solution of H_2_O_2_ (30%, VWR International, CAS: 470301-280) was spiked at known concentrations into the minimal tryptic soy broth (TSB)/casaminoacid (CAA) medium used for all experiments (1% tryptic soy broth and 0.5% casamino acids). A 25-μm diameter Pt UME was used for all experiments as the working electrode. Since H_2_O_2_ oxidation is an inner sphere reaction on an electrode surface, we set a 15-min stabilization time with the UME held at +0.5 V versus Ag/AgCl reference to allow for UME surface equilibration with medium. All experiments were done at 31°C to minimize evaporation.

### Growth curve experiments.

All bacterial strains were grown on tryptic soy agar yeast extract (TSAYE) plates overnight, and then colonies were picked for next-day overnight growth in tryptic soy broth yeast extract (TSBYE). The stationary-phase bacteria were then back diluted to an optical density at 600 nm (OD_600_) of 0.05 and grown to mid-log phase (~OD_600_ = 0.5) in TSBYE. At mid-log phase, cells were once again diluted to an OD_600_ of 0.05 and cell density (OD_600_) was measured over time in the minimal TSB/CAA medium. Optical density was measured using a Biochrom WPA Biowave II UV/Vis spectrophotometer.

### Bacterial strains.

Aggregatibacter actinomycetemcomitans VT1169 (58), Streptococcus gordonii DL1.1 (ATCC 49818), and wild-type Pseudomonas aeruginosa (PA14) were used for all experiments as the wild-type strains. Aggregatibacter actinomycetemcomitans *katA*^−^ was obtained from an ordered Tn-seq library (59).

### Biofilm chronoamperometry.

Bacteria were grown on a TSAYE plate overnight, and the next day, colonies were grown overnight in TSBYE. Cells were back diluted in the morning to an OD_600_ of 0.1 and were grown to a final OD_600_ of ~0.6 to 1. Bacterial culture was spun down and washed once in TSB/CAA. Cell densities were then standardized to a final OD_600_ of 10. A 1:1 ratio of resuspended cells to 3% noble agar was added into the 2 mm well of the stainless-steel disc, and the solution was pipetted immediately up and down once, then covered with a glass slide, and placed at 4°C for 10 to 15 min to allow the agar to solidify. On occasions when the cells and noble agar did solidify fast, which was evident by pipetting, we cleaned the stainless-steel disc to remove all cells and noble agar and attempted to form biofilms again. The stainless-steel disc was removed and then attached to the heating chamber with double-sided tape, and the electrochemical cell was assembled and leveled using a bubble level. After the approach curve to the surface, placing the UME tip 100 μm from the surface of the disc, cells were heated for 15 min to bring the temperature up to 31°C while the UME stabilized as described for obtaining the calibration curve. After the 15 min, the UME remained poised at +0.5 V versus Ag/AgCl and scanned along the z-plane while measuring H_2_O_2_ current.

### Biofilm chronopotentiometry.

We followed the protocol similarly to how we followed that of the chronoamperometry experiments, but to ensure we could measure the potential above the biofilm, we resuspended the cells to an OD_600_ of 100 before adding a 1:1 ratio of cells to 3% Noble agar. For coculture experiments, the cell numbers remained the same, and the bacteria were mixed at a 1:1 ratio while maintaining a final overall OD_600_ of 100. Furthermore, the cells were grown for a longer time in the agar biofilm, and the setup was heated for 2 h at 31°C. The software requires the user to set two experimental periods, the induction period and the electrolysis period. We set the induction period as having a 10-minute stabilization time with the UME set to maintain a current of 0.5 nA, and during this period no data were collected. During the electrolysis period, the UME was still set to maintain a current of 0.5 nA with all parameters kept the same, but the instrument recorded data. No scanning occurred during the induction period, while the electrolysis period coincided with the UME measuring potential while scanning in the z-plane. A current of 0.5 nA was chosen as a reasonably low current based on the calibration curve presented in Fig. S6. The instrument and software operated with the IUPAC standard, and therefore the 0.5 nA current that was applied is considered anodic.

Speeds for both experiments were 100 μm/s for the *x* axis and 50 μm/s for the *y* axis as noted in the SI script: raster scanning for micromanipulator (C#).

### Mapping potentials above biofilms.

Initially, a shift to higher potentials was observed over time when the UME was positioned away from the biofilm, likely due to absorbed species fouling the surface. Data from these control experiments significantly complicated analysis of the data acquired above the biofilm, necessitating that we conceptually approached data analysis and image processing in a manner different from that used previously. We began by rewriting the Nernst equation (equation 2) as follows:

(2) $$E=E^{0}-\frac{\mathrm{RT}}{\mathrm{nF}}\ln\left(\frac{\left[\mathrm{Red}\right]}{\left[\mathrm{Ox}\right]}\right)$$

(3) $$E-E^{0}=-\frac{\mathrm{RT}}{\mathrm{nF}}\ln\left(\frac{\left[\mathrm{Red}\right]}{\left[\mathrm{Ox}\right]}\right)$$

Here, *R* = 8.3145 J/mol K, T = 305 K, *n* = 1, and *F* = 96,485.309 C/mol. Therefore,

(1) $$\frac{E-E^{0}}{-0.0263\text{ V}}=\ln\left(\frac{\left[\mathrm{Red}\right]}{\left[\mathrm{Ox}\right]}\right)$$

Here, *E* is the potential measured, *E*^0^ is the average potential of medium control biofilm scans, and [Red]/[Ox] corresponds to the relative activity of reduced species to that of oxidized species. Due to the assumptions that go into deriving the Nernst equation, and since it is not an electrochemical technique-specific expression, we treat it as a general electrochemical expression when applying it to all chronopotentiometry experiments. Since many molecular redox processes are involved when measuring the potential, we consider the relative activity of oxidized species to that of reduced species rather than concentrations. Additionally, our experiments have an [Ox]/[Red] greater than one, and thus we have taken this for easier visualization. For image processing, we adapted a Python script that would perform baseline subtraction to correct for fouling, and indirectly, this served to set up equation 1 as *E *− *E*^0^, which was solved for by the subtraction. [Ox]/[Red] was solved for using the same equation.

### Measuring H_2_O_2_ consumption rates.

Cells were prepared as described above for chronoamperometry experiments since these experiments were performed using only A. actinomycetemcomitans biofilms, and we were interested in knowing the immediate response to H_2_O_2_. Cells were first submerged in 5 mL of minimal TSB/CAA medium, heated to 31°C for 15 min, and then flooded with 5 mL of ~9 to 10 mM H_2_O_2_ solution, and the current response was measured by holding the UME at +0.5 V versus the Ag/AgCl reference. To analyze the maximum, minimum, and optimal consumption rates, we used the R package RespR.

### l-Lactate and H_2_O_2_ contribution to redox potential.

For pH measurements, we used an UltraBASIC pH meter (Denver Instrument, UB-5) and added known concentrations of l-lactate (Alfa Aesar, CAS: 79-33-4) to TSB/CAA medium. The redox potential changes upon adding l-lactate and H_2_O_2_ were measured in the same setup used for chronopotentiometry experiments; the TSB/CAA medium was heated for 10 min at 31°C with the UME set to maintain a current of 0.5 nA, and this current was maintained past the induction period throughout the electrolysis period. The potential was measured and then averaged for ~20 s at the beginning (blank) and then measured and averaged for ~20 sec for each condition. The averaged blank potential was then subtracted from the averaged potential measured for each condition and the [Ox]/[Red] was solved for using equation 1.

### Statistical analysis.

Analysis of variance (ANOVA) was conducted in Excel and then repeated using astatsa.com along with *post hoc* Tukey honestly significant difference (HSD) test.
