# Supplementary material for: Development of a Versatile, Low-Cost Electrochemical System to Study Biofilm Redox Activity at the Micron Scale
Source: Appl Environ Microbiol. 2022 Jun 27;88(14):e00434-22. doi: 10.1128/aem.00434-22 (PMC9328185; doi:10.1128/aem.00434-22)
Supplement: Supplemental file 1 — Supplemental Materials and Methods, Fig. S1 to S13, and Tables S1 to S4. Download aem.00434-22-s001.pdf, PDF file, 2.4 MB [file aem.00434-22-s001.pdf]

Materials and Methods

Script: Raster Scanning for Micromanipulator (C#).

```
using System;
using Zaber.Motion;
using Zaber.Motion.Ascii;

// namespace name may differ in your code
namespace Raster_Mode
{
    class Program
    {
        static void Main(string[] args)
        {
            Library.EnableDeviceDbStore();

            // The rest of your program goes here
            using (var connection = Connection.OpenSerialPort("COM3"))
            {
                var deviceList = connection.DetectDevices();
                Console.WriteLine($"Found {deviceList.Length} devices.");

                // The rest of your program goes here

                var device = deviceList[1];
                var axis1 = device.GetAxis(1);
                var axis2 = device.GetAxis(2);
                var axis3 = device.GetAxis(3);

                // Speed of X-Axis
                axis1.Settings.Set("maxspeed", 100, Units.Velocity_MicrometresPerSecond);
                var speedY = axis1.Settings.Get("maxspeed", 
Units.Velocity_MicrometresPerSecond);

                // Speed of Y-Axis
                axis2.Settings.Set("maxspeed", 50, Units.Velocity_MicrometresPerSecond);
                var speedX = axis2.Settings.Get("maxspeed", 
Units.Velocity_MicrometresPerSecond);

                // Speed of Z-Axis
                axis3.Settings.Set("maxspeed", 50, Units.Velocity_MicrometresPerSecond);
                var speedZ = axis3.Settings.Get("maxspeed", 
Units.Velocity_MicrometresPerSecond);



                Console.WriteLine("Maximum speed Y [um/s]: {0}", speedY);
                Console.WriteLine("Maximum speed X [um/s]: {0}", speedX);
                Console.WriteLine("Maximum speed Z [um/s]: {0}", speedZ);


                //Write Time Experiment Started

                //DateTime now = DateTime.Now;
                Console.WriteLine(DateTime.Now);
                //Console.ReadLine();

                // Scanning
```

```
                //Line 2 makes it a bit easier to change the number of iterations
                int iterations = 12;
                for(int i =0; i < iterations; i++)
                {
                    Console.WriteLine("Iteration: " + i + " Time: " +
DateTime.Now.ToShortTimeString() + " Y forward");
                    axis2.MoveRelative(6500, Units.Length_Micrometres);

                    Console.WriteLine("Iteration: " + i + " Time: " +
DateTime.Now.ToShortTimeString() + " X forward");
                    axis1.MoveRelative(300, Units.Length_Micrometres);

                    Console.WriteLine("Iteration: " + i + " Time: " +
DateTime.Now.ToShortTimeString() + " Y backward");
                    axis2.MoveRelative(-6500, Units.Length_Micrometres);

                    Console.WriteLine("Iteration: " + i + " Time: " +
DateTime.Now.ToShortTimeString() + " X forward");
                    axis1.MoveRelative(300, Units.Length_Micrometres);

                };


                //Write Time Experiment Ended
                Console.WriteLine(DateTime.Now);


                //Extra Moves
                //1.) Move to the 10mm position
                //axis1.MoveAbsolute(10, Units.Length_Millimetres);

                //2.) Home Axis
                //axis1.Home();

            }

        }
    }
}
```

Script: Leveling Chronopotentiometry Data (Python).

```
import os
import pandas as pd
import matplotlib.pyplot as plt
import numpy as np
import csv
from scipy.sparse.linalg import spsolve
from scipy import sparse


def baseline_als(y, lam=1e8, p=0.0001, niter=10):
    """
     Purpose:
        Baseline correction using asymmetrically
     Input:
         y -- List of n entries of y axis variable
         lam -- Lambda affects smoothness (Recommended: 10^2 ≤ λ ≤ 10^9)
         p -- will affect asymmetry (Recommended: 0.001 ≤ p ≤ 0.1)
         niter -- Iterations of smoothing (Recommended 10-100) (WARNING: WILL AFFECT
RUNTIME SUBSTANTIALLY)
     Output:
        z -- List of n entries of baseline offset
     Reference:
        https://bit.ly/314Tku3
    """
    L = len(y)
    D = sparse.csc_matrix(np.diff(np.eye(L), 2))
    w = np.ones(L)
    for i in range(niter):
        W = sparse.spdiags(w, 0, L, L)
        Z = W + lam * D.dot(D.transpose())
        z = spsolve(Z, w * y)
        w = p * (y > z) + (1 - p) * (y < z)
    return z


def display_data(x, y, title="Untitled"):
    """
     Purpose:
        Display input data
     Input:
         x -- List of n entries of x axis variable
         y -- List of n entries of y axis variable
         title -- String to designate the title of the displayed graph
         style -- ('Scatter' or 'Plot') format in which data is displayed
     Output:
        N/A
    """
    plt.figure(figsize=(10, 10))
    plt.style.use('seaborn')
    plt.scatter(x, y, marker=".", s=100, edgecolors="black", c="red")
    plt.title(title)
    plt.show()


def run():
```

```
    """
     Purpose:
        Main logical method - Keeps the main method clean
     Input: - N/A
     Output: - N/A
    """
    # Read in Data
    var = pd.read_excel('Sample.xlsx')
    x = list(var['Time'])
    y_original = list(var['Volts'])

    # Calculate baseline and subtract from y data
    baseline = baseline_als(y_original)
    y_flattened = y_original - baseline

    # Display data
    display_data(x, y_original, 'Original Data')
    display_data(x, y_flattened, 'Modified Data')

    # Data processing pre-saving
    save_data = np.array([x, y_flattened])
    save_data = np.transpose(save_data)

    # Save Output data to CSV
    f = open(os.getcwd() + '\\modified_data.csv', 'w')
    writer = csv.writer(f)

    for row in save_data:
        writer.writerow(row)

    f.close()


if __name__ == '__main__':
    run()
```

Script: Converting Scanning to Image (R).

```
rm(list=ls())

library(tidyr)
library(dplyr)
library(plyr)
library(plot3D)
library(data.table)
library(rgl)
library(plotly)
library(colorRamps)
library(ggpubr)
library(ggplot2)


Data <- read.csv("ScanningData.csv")

# 1.) Reminder, Set Zero Point! Manually subtract.

# 2.) Specify Parameters

# 2a.) Calibration Curve
y = Data$i
m = 1
b = 0

# 2b.) Scanning in X, microns/sec and microns respectively
ssx = 50
sdx = 6500
stx = sdx/ssx

# 2c.) Scanning in Y, microns/sec and microns respectively
ssy = 100
sdy = 300
sty = sdy/ssy

# 3.) Adjust current to concentration via calibration curve

C = (y-b)/m

DataN <- cbind(Data,C)

# 4.) Adjust X and Y values while removing gaps

# 4a.) Cut into lines
#d <- split(DataN, rep(1:ceiling(nrow(DataN)/chunk), each=chunk, length.out=nrow(DataN)))

chunk <- (stx+sty)*10
n <- nrow(DataN)
r <- rep(1:ceiling(n/chunk),each=chunk)[1:n]
d <- split(DataN,r)

# 4b.) Remove last 100 points and add column with Y values
```

```
lp = chunk-sty*10
d2 = lapply(d, function(x){x[1:lp,]})

# 4c.) Take the Time.SA and standardize it by subtracting first value from all

d3 = sapply(1:length(d2), function(i_x){
  x = d2[[i_x]]
  temp.SA = x$Time.SA
  temp.SA = temp.SA - temp.SA[1]

  ret = x
  ret$Time.SA = temp.SA

  if(i_x%%2){ # mod2 is equivalent to asking "is this odd"
    ret$X.SA = temp.SA * ssx # in microns
  }else{
    # if it's even, aka i_x%%2 = 0, then we flip
    ret$X.SA = rev(temp.SA * ssx) # in microns
  }

  # add y's
  ret$Y.SA = (as.numeric(i_x)-1)*sdy

  return(list(ret))
})

head(d3[[3]])

valx <- length(d3)
d4 <- d3[-valx]

df.all = do.call('rbind', d4) # combines all data frames in list by row

# 5.) Plotting Data
x <- df.all$X.SA
y <- df.all$Y.SA
z <- df.all$C

scatter3D(x, -y, z, phi = 30, bty = "g", type = "l",
          ticktype = "detailed", lwd = 4)

fig <- plot_ly(x = df.all$X.SA,
               y = df.all$Y.SA,
               z = df.all$C,
               intensity = z,
               colorscale = "Rainbow",
               cauto = F,
               cmin = -1,
               cmax = 4.5,
               type = 'mesh3d')%>%
  #hide_colorbar()%>%
  layout(scene = list(xaxis = list(title ="X-Distance (\u03BCm)", showgrid = FALSE, dtick
= 1000, range = c(1, 6000)),
```

```
                      yaxis = list(title ="Y-Distance (\u03BCm)", showgrid = FALSE, range
= c(1, 6000)),
                      zaxis = list(
                        range=c(0, 4.5),
                        constrain="domain",
                        title = "[H<sub>2</sub>O<sub>2</sub>] (mM)"),
                      font=list(size = 12)) )
fig
```

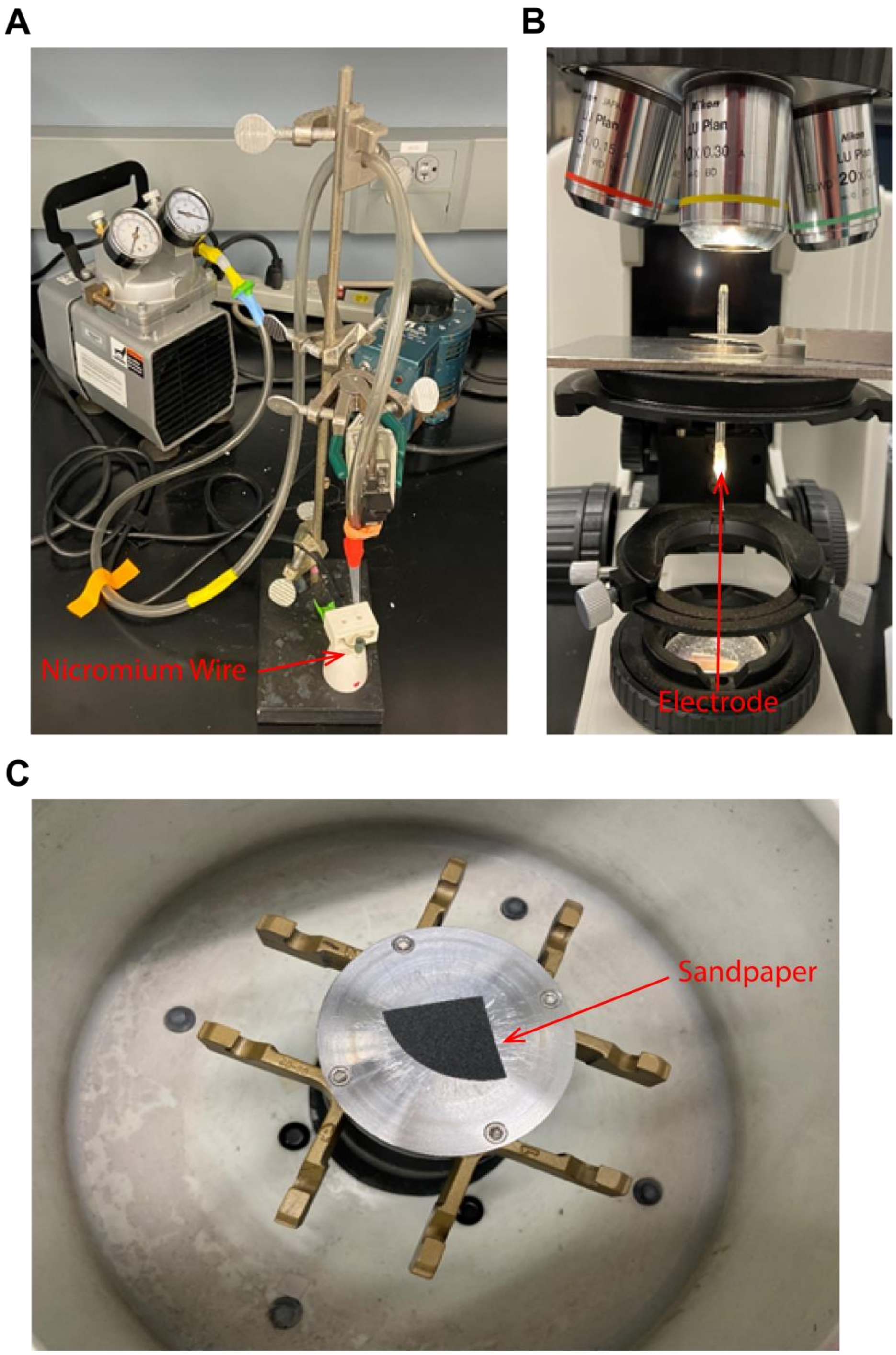

FIG S1 UME fabrication equipment. **(A)** Electrode sealing apparatus. Electrode sealing requires a nickel chromium wire to be heated via direct current. This is achieved using a Variac device set to no more than 10 V. The ceramic spacer is used to keep the coil in place. Upon heating, the vacuum pump is turned on which is connected to a tube that terminates with an adapter made from a 1 ml pipette tip and hangs attached to a sawed pipettor to allow for vertical positioning of the electrode. **(B)** The electrode surface can be visualized using a microscope where the stage has been modified to allow for the electrode to hang from reverse action tweezers. **(C)** Modified

polishing wheel. Polishing of the electrode after sealing was done using a modified centrifuge. Certain speeds may be more comfortable for users, and there are several considerations: water pooled during polishing must be pumped out when necessary, the “turn table” portion in the middle where sandpaper with a PSA back is stuck must be balanced, and the sandpaper grit must be switched out frequently (180, 600, then 1200 grit; usually one quarter of one disc was enough to polish an electrode once).

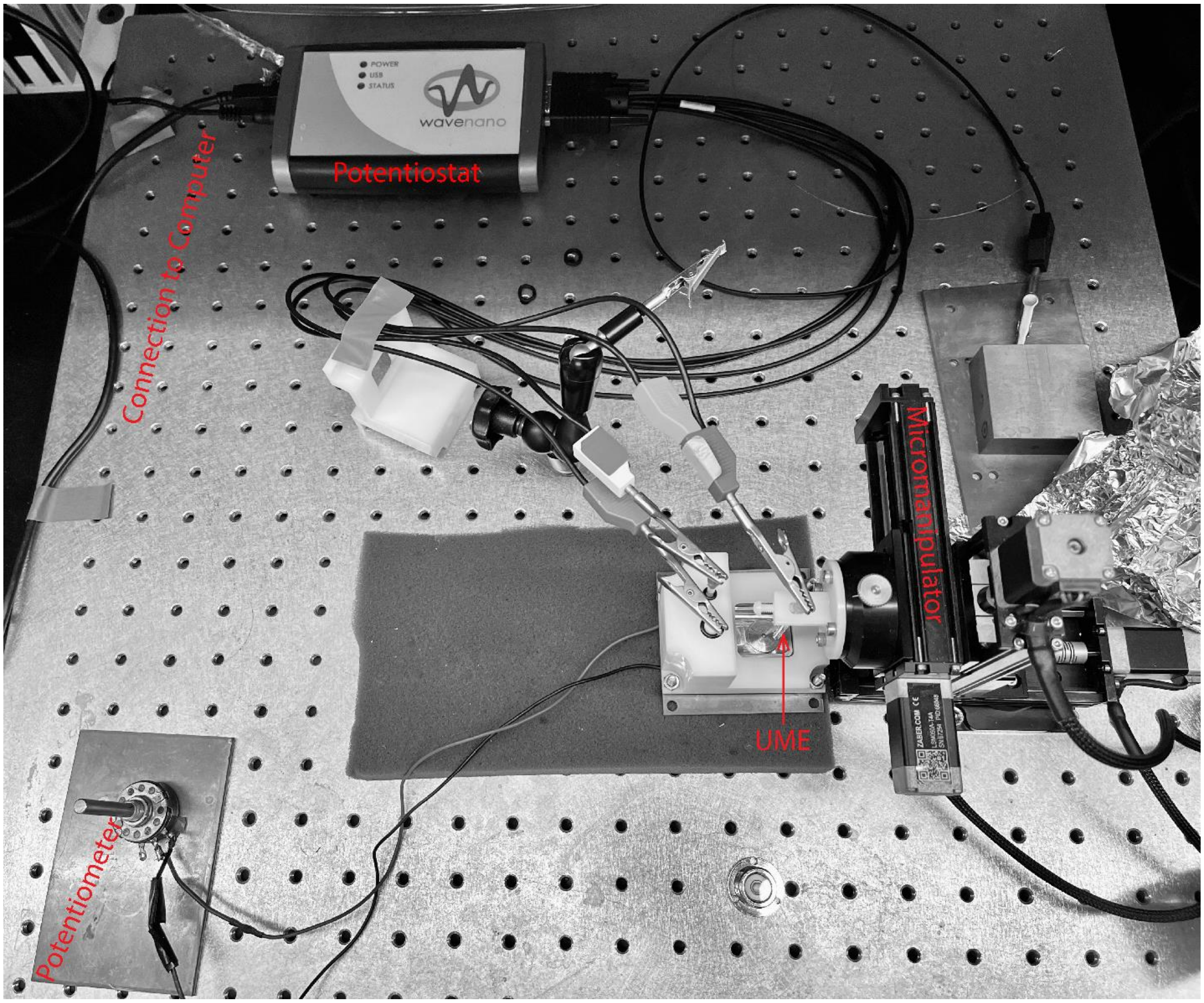

FIG S2 Photograph of the electrochemical setup as illustrated in Fig 1A.

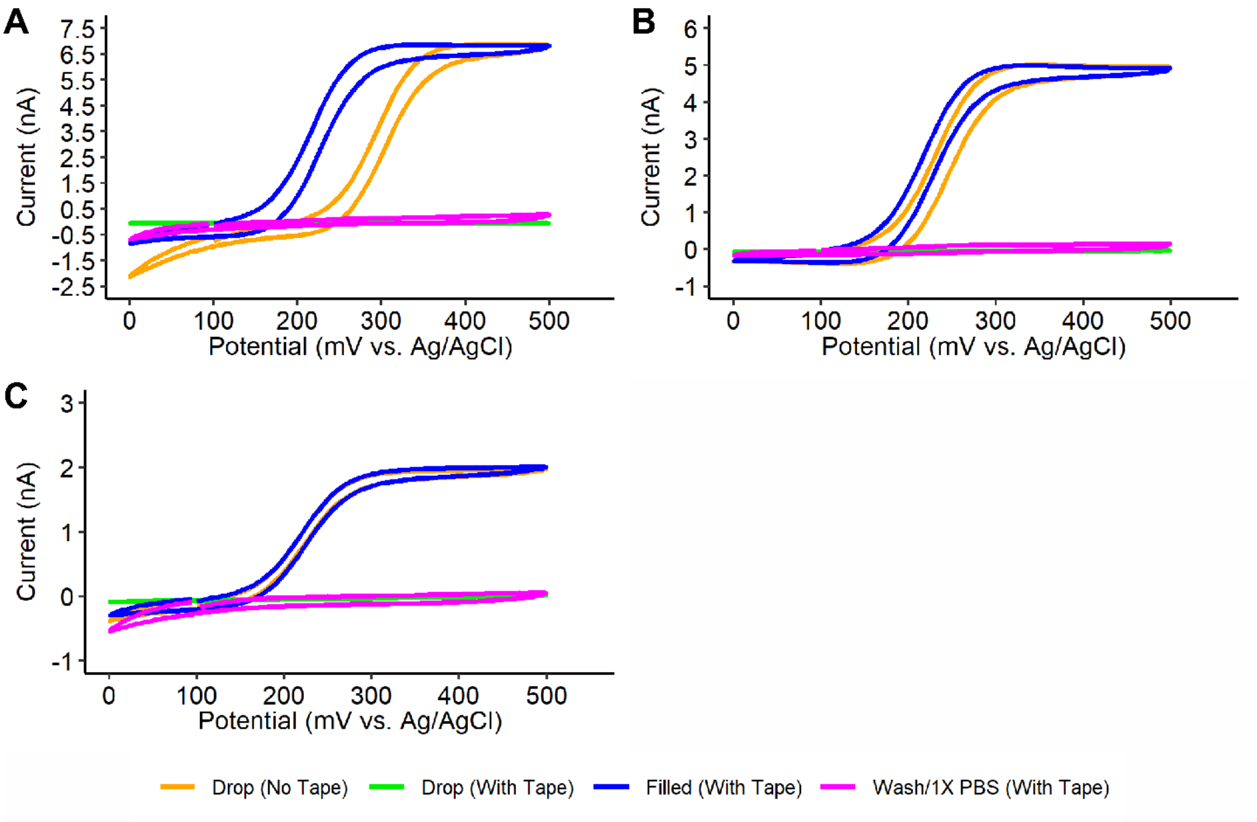

FIG S3 Control experiments using the electrochemical system. Various electrodes were used: **(A)** 25 µm platinum, **(B)** 25 µm gold, and **(C)** 10 µm platinum. The orange line represents the cyclic voltammetry experiment resulting from a drop of 1.5 mM FcMeOH on the stainless-steel disc. The green line represents the current when the electrode is cycled in a 1.5 mM FcMeOH drop on the insulating tape. The blue line represents the electrochemical cell with tape when the entire cell is flooded with 1.5 mM FcMeOH (positive control). Finally, the magenta line represents cycling the electrode when the entire cell with tape is washed three times with 1X PBS to remove FcMeOH, and then filled entirely with 5 mL 1X PBS (negative control). The second replicate is shown in **Fig. 1C**.

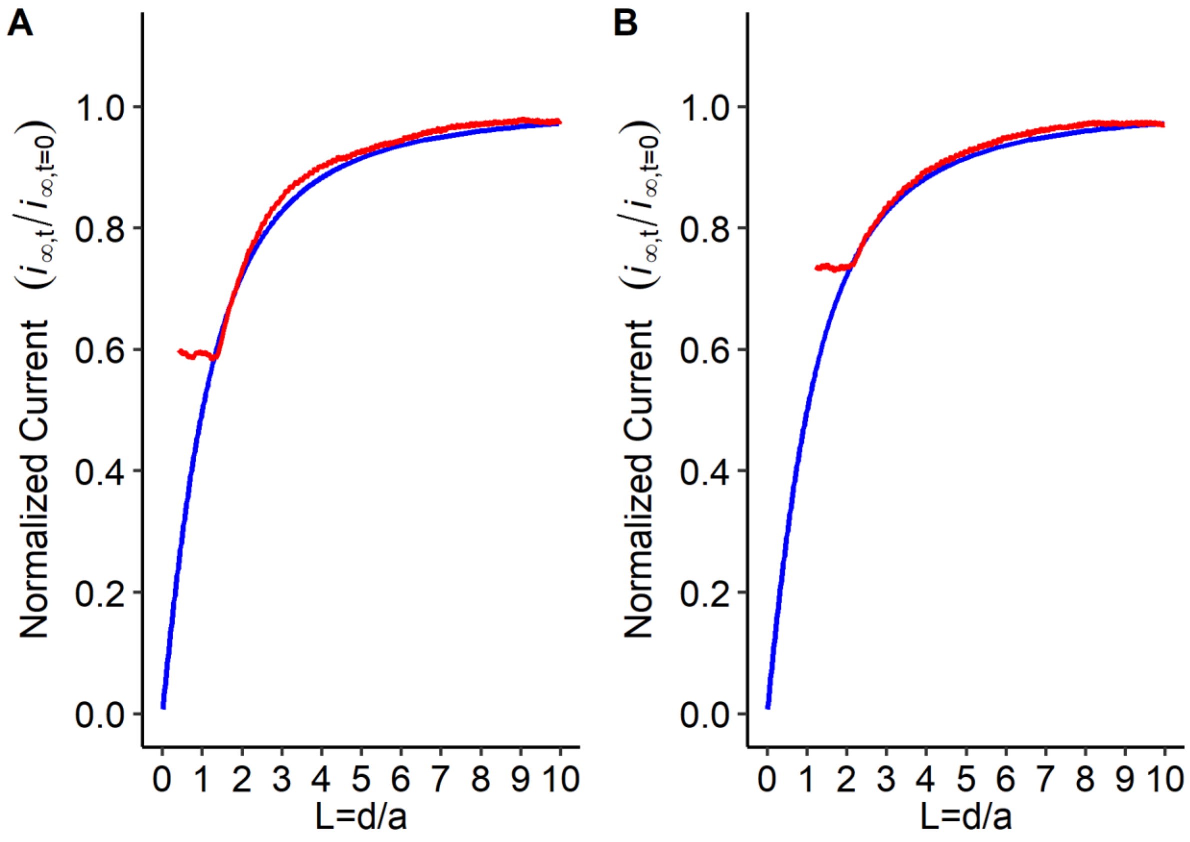

FIG S4 Approach curves to the stainless-steel disc surface. **(A)** The first approach was done to the metal surface which resulted in a 60% decrease in current (this figure is also shown in **Fig. 1D** and is provided here for context). **(B)** The UME was then raised 100 µm and a second approach curve performed, which resulted in a 75% decrease in current. The red line corresponds to the acquired data with the blue line representing the theoretical approach of an electrode. The UME was a 25 µm diameter gold UME with an RG=10. Of note, we have left the red trail visible when the UME was approached and then stopped to illustrate z-axis stability.

A

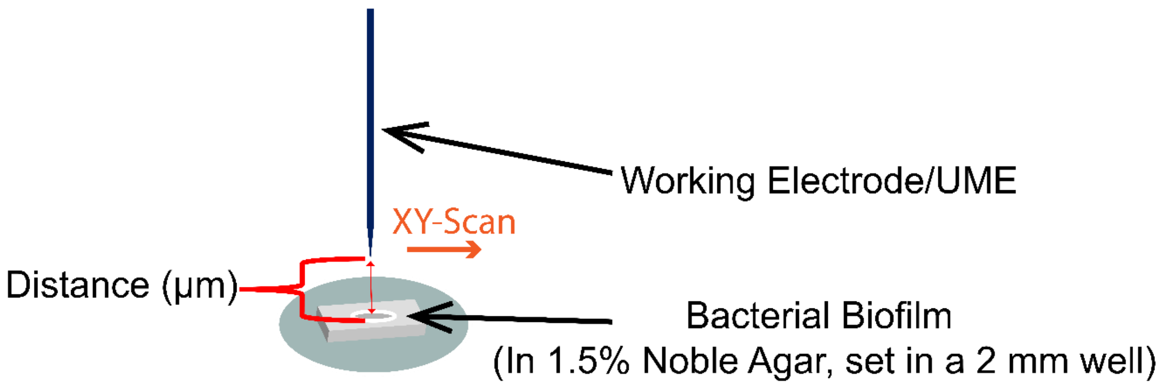

B

Electrode	25 µm Au	25 µm Pt	10 µm Pt
Start	25.13 µm<br>33.13 µm	21.13 µm<br>27.13 µm	8.95 µm<br>11.45 µm
Max	57.13 µm<br>72.13 µm	184.63 µm<br>227.13 µm	22.85 µm<br>28.85 µm
Min	2.63 µm<br>3.38 µm	0 µm<br>0.13 µm	8.65 µm<br>11.15 µm

FIG S5 Scanning experimental setup. **(A)** Scanning along the surface of the Noble agar without bacteria yielded a relatively flat surface. The UME approached the surface using a 'drop' approach curve and scanned along the XY-plane. **(B)** The highest and lowest points correspond to a single µm point where the distance was measured. For these measurements we approached the electrode within the hindered diffusion region and scanned along the y-axis at 5 µm/sec for 15-20 minutes which corresponds to a scan of 4.5-6 mm along the stainless steel disc including a blank biofilm in the center. Please note, the starting distance is within the hindered diffusion region where it is appropriate to measure the evenness of the surface. Maximum values usually corresponded to extreme edges of the stainless steel disc.

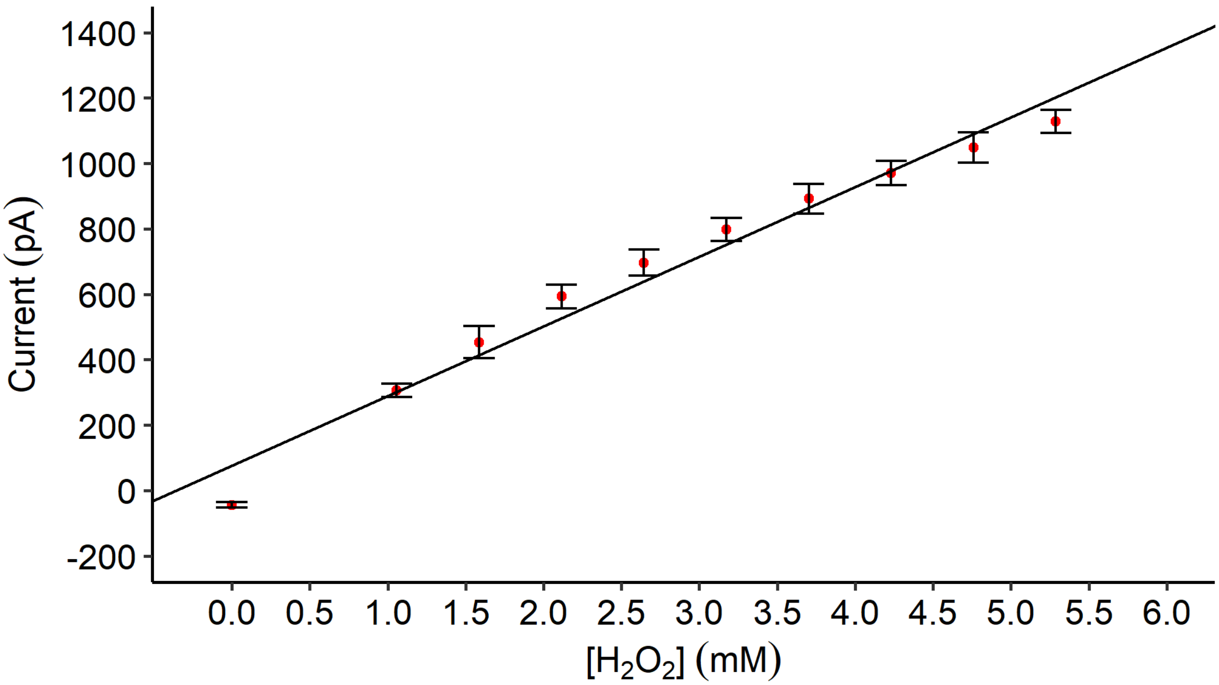

FIG S6 H_2O_2 Calibration curve performed in the absence of cells. Current was measured for H_2O_2 at 31ºC in TSB/CAA media with a 15-minute stabilization time using a UME held at +0.5 V vs Ag/AgCl. Measurements were taken on three different days with three different 25 µm Pt diameter UMEs (RG ~8). Here the line is fit to the equation i = 212.77355*[H_2O_2] + 76.62113 with an R^2 value of 0.9718. A linear current response is expected for microelectrodes given the current response is directly proportional to concentration for microelectrodes ($i = 4n\mathcal{F}cda$; i = current, n = number of electrons involved in the redox reaction, $\mathcal{F}$ = Farday constant, c = concentration of redox molecule in solution, d = diffusion constant of molecule at that temperature, and a = microelectrode radius). Error bars represent ± standard deviation (n=3).

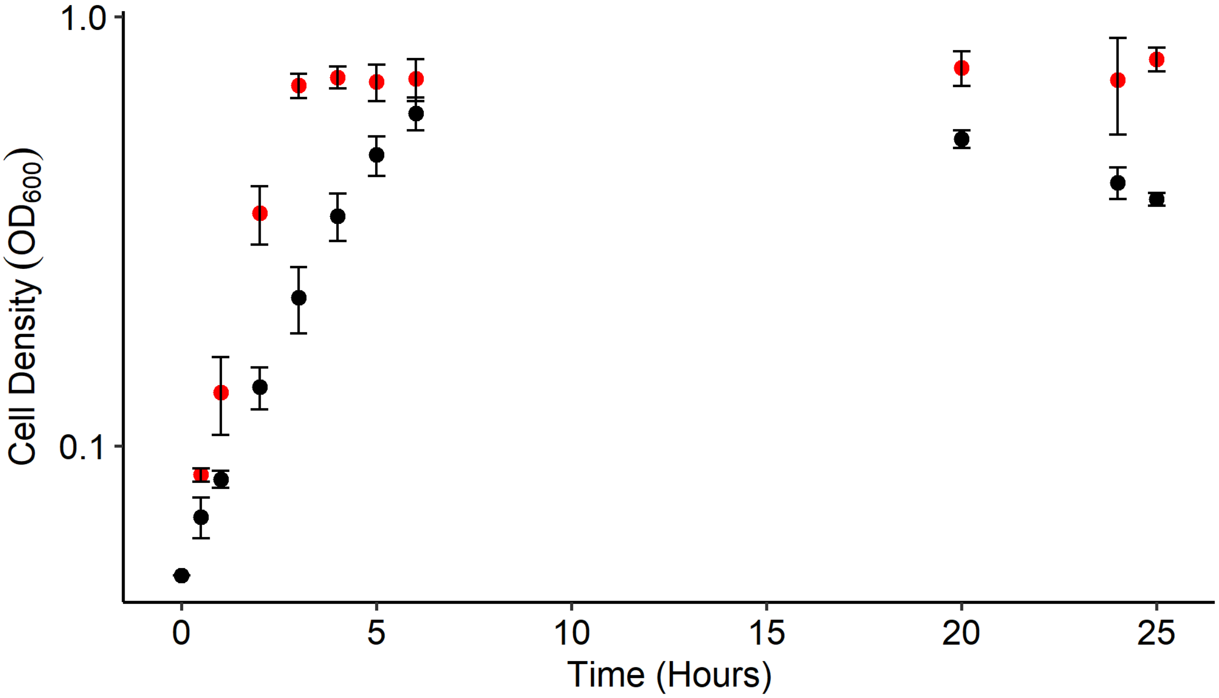

FIG S7 *A. actinomycetemcomitans* and *S. gordonii* growth curves in TSB/CAA media. Red corresponds to *S. gordonii* growth and black corresponds to *A. actinomycetemcomitans* growth where error bars represent ± one standard deviation (n=3).

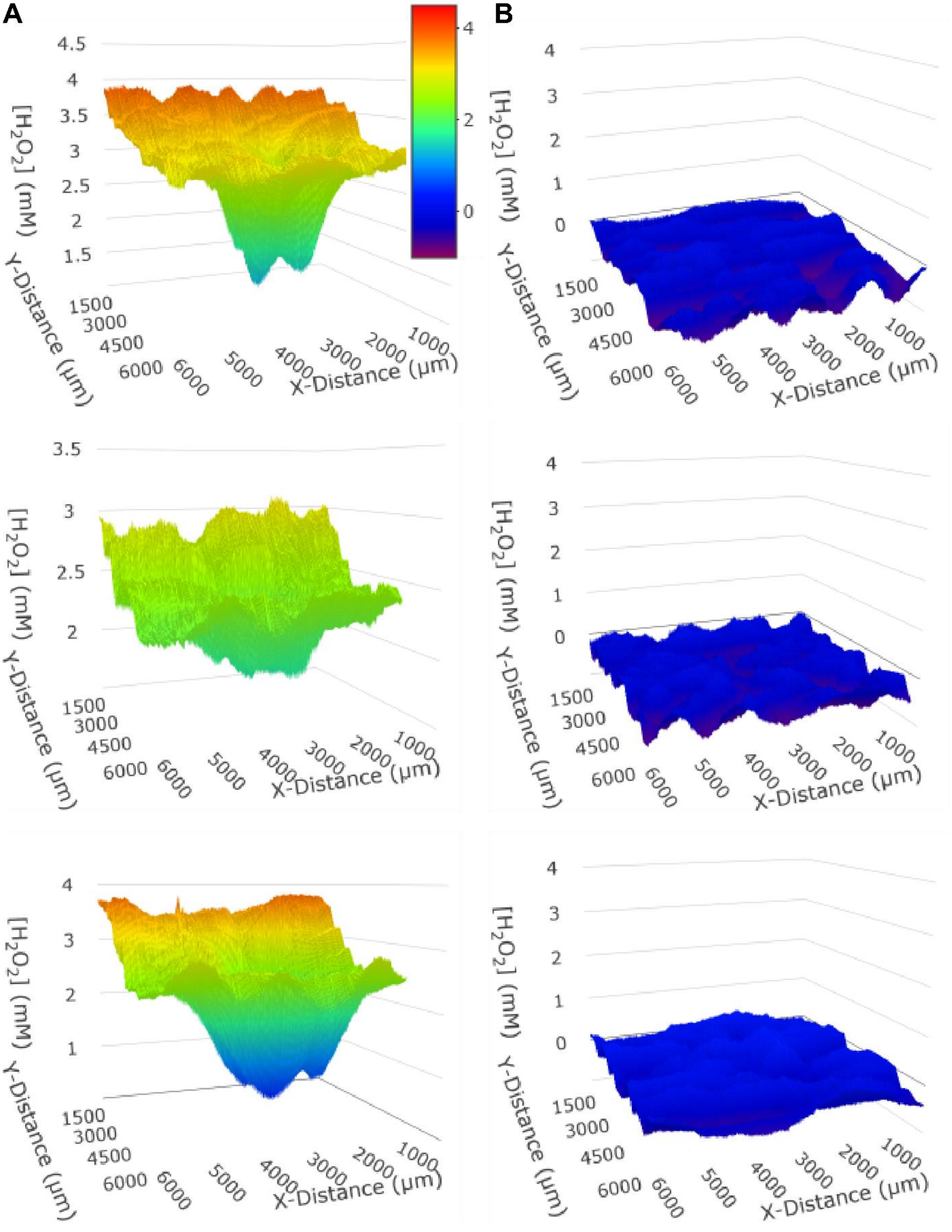

FIG S8 H_2O_2 detoxification profiles 100 µm above biofilms. Shown are three replicates for **(A)** wt *A. actinomycetemcomitans* with H_2O_2 added and **(B)** wt *A. actinomycetemcomitans* with no H_2O_2 added. All data in the first row are the representative experiments shown in **Fig. 2**.

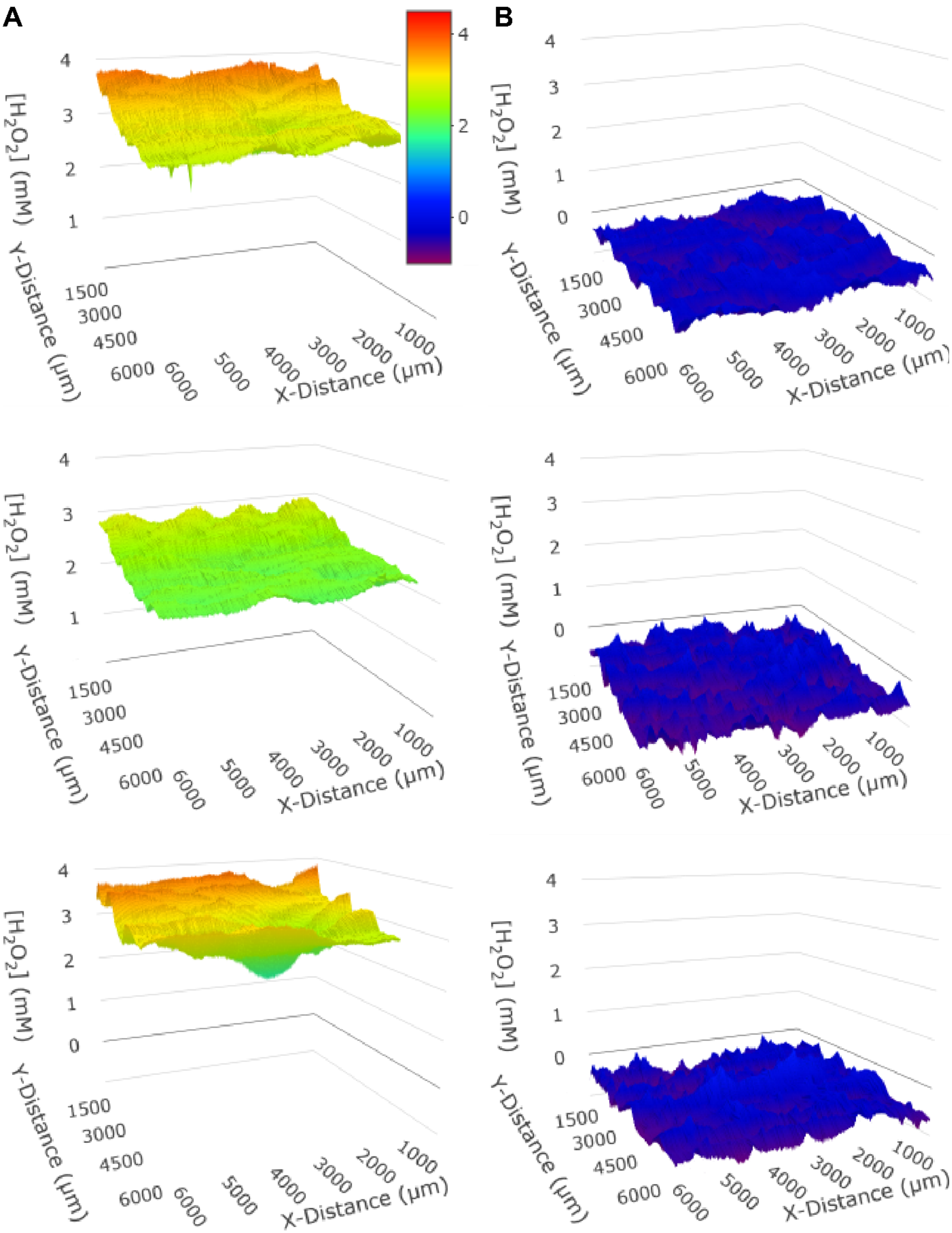

FIG S9 H_2O_2 detoxification profiles 100 µm above biofilms. Shown are three replicates for **(A)** *A. actinomycetemcomitans katA*⁻ with H_2O_2 added and **(B)** *A. actinomycetemcomitans katA*⁻ with no H_2O_2 added. All data in the first row are the representative experiments shown in **Fig. 2**.

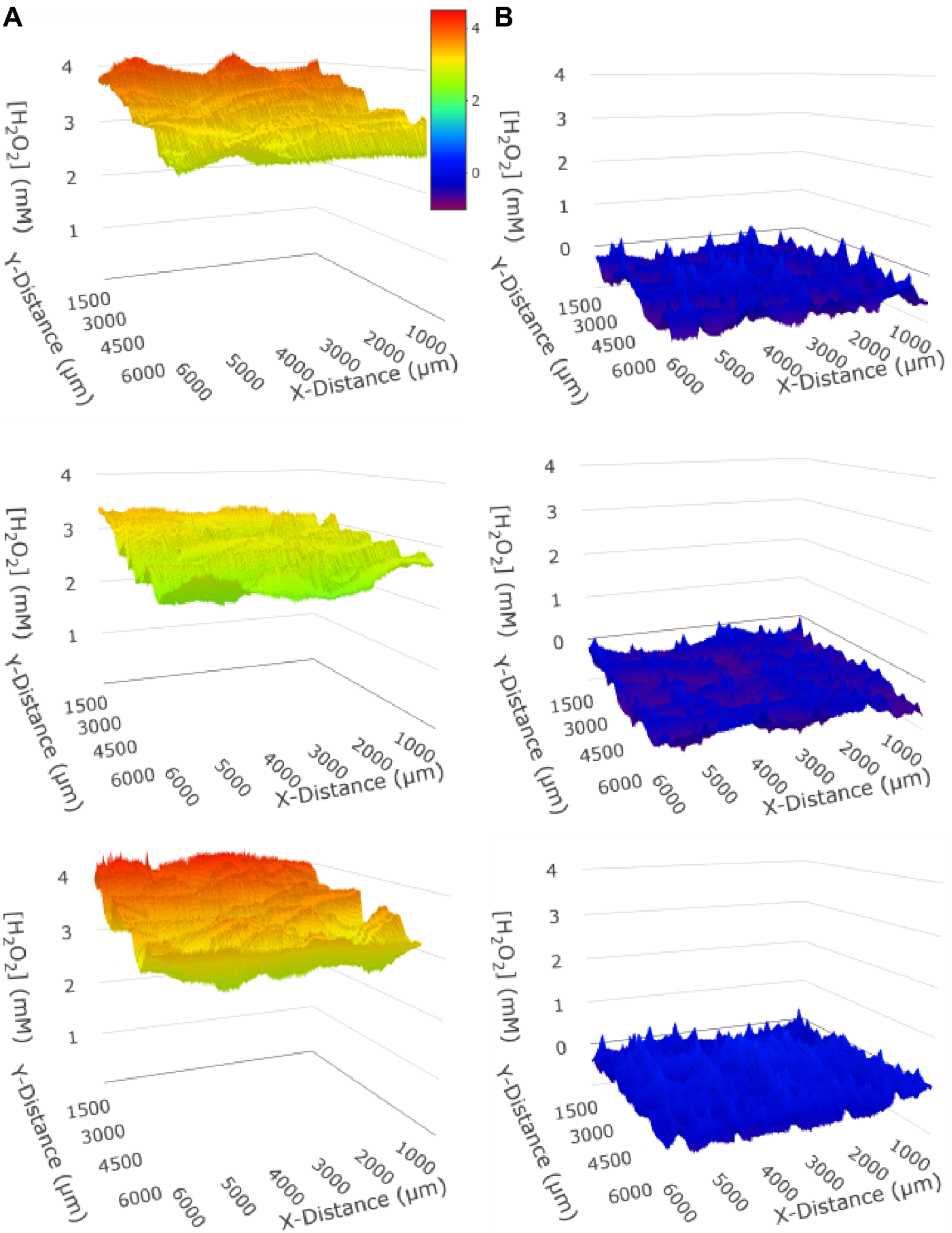

FIG S10 Controls for H_2O_2 detoxification profiles. Shown are three replicates for **(A)** media with H_2O_2 added and no bacteria **(B)** media with no bacteria or H_2O_2. All data in the first row are the representative experiments shown in **Fig. 2**.

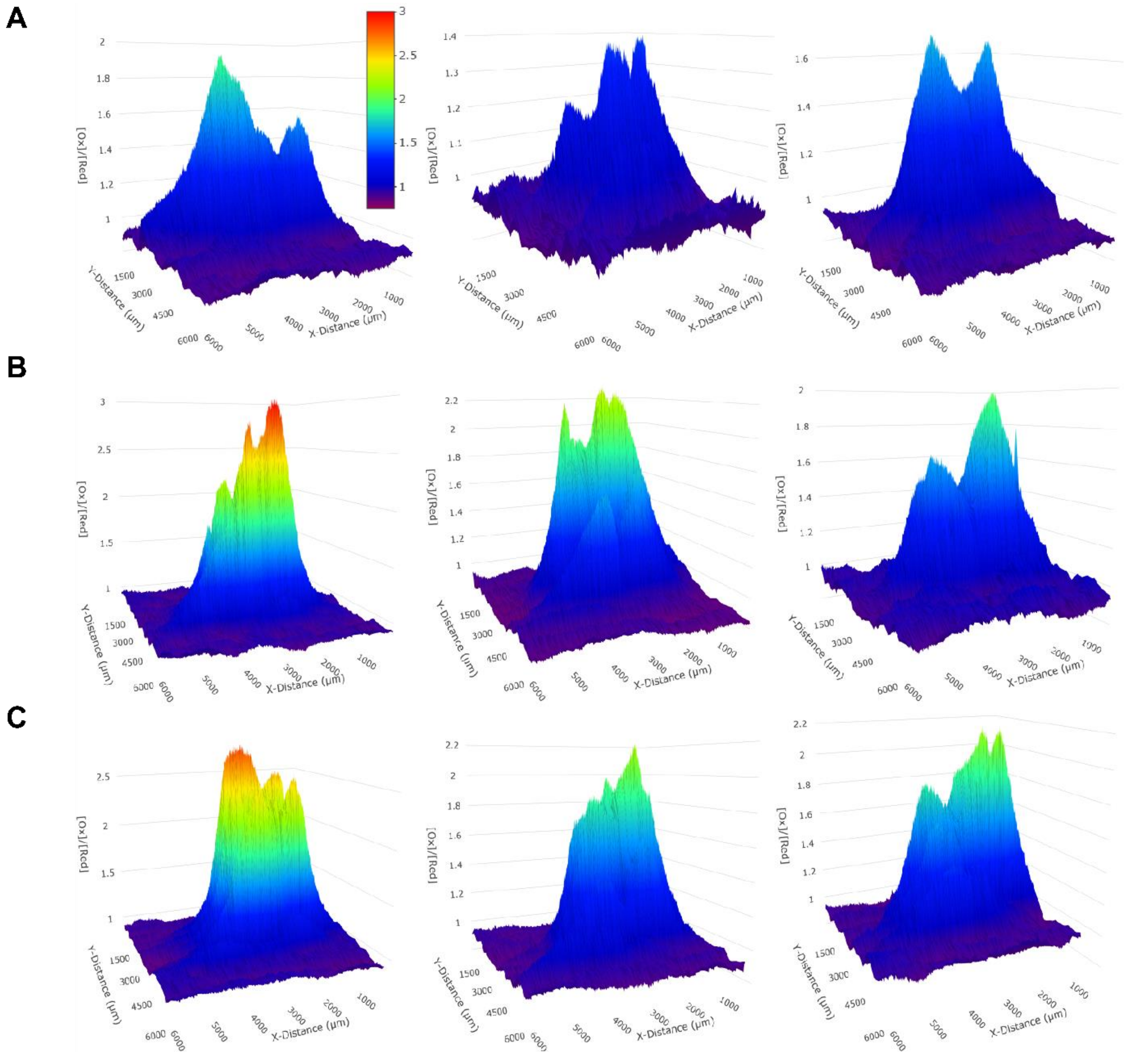

FIG S11 All chronopotentiometry scans 100 µm above biofilms. **(A)** wt *A. actinomycetemcomitans* biofilms, **(B)** *S. gordonii* monoculture biofilms, and **(C)** *A. actinomycetemcomitans wt/S. gordonii* biofilms. All data in the first column are representative experiments shown in **Fig. 4**.

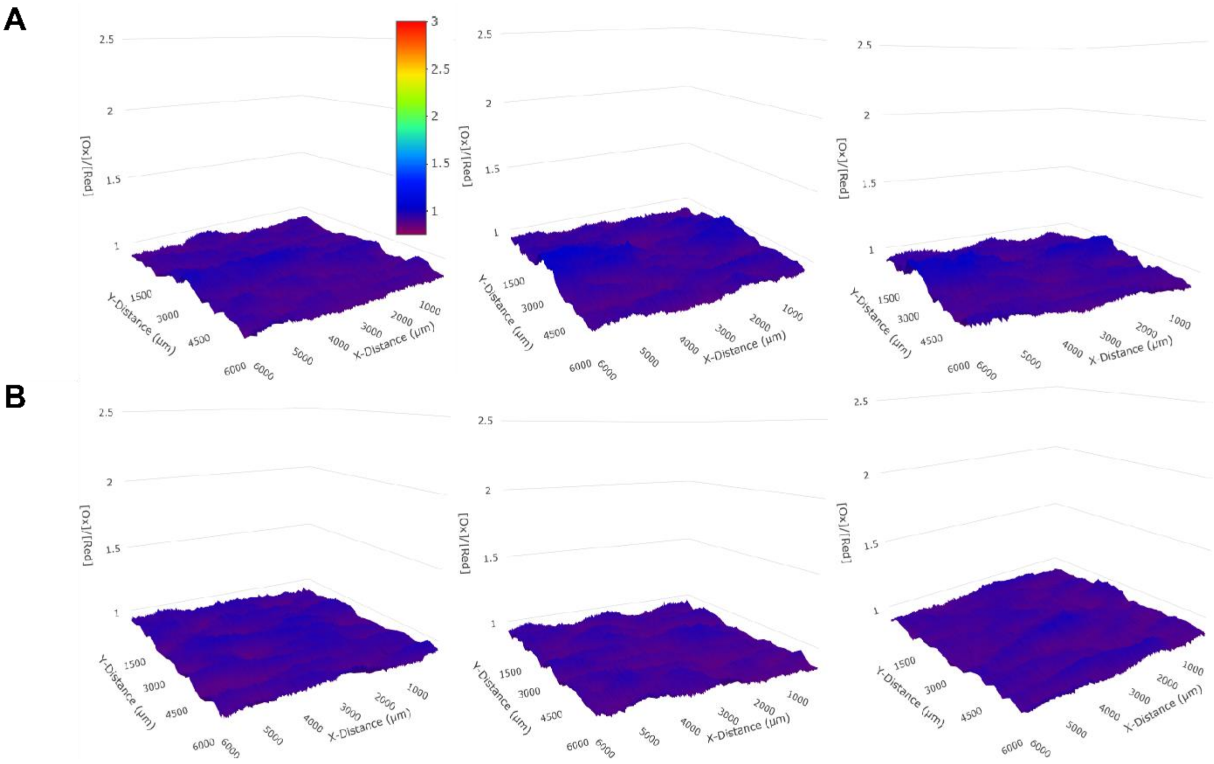

FIG S12 All chronopotentiometry scans 100 µm above **(A)** *P. aeruginosa* biofilms and **(B)** media with no bacteria. All data in the first column are replicates shown in **Fig. 4**.

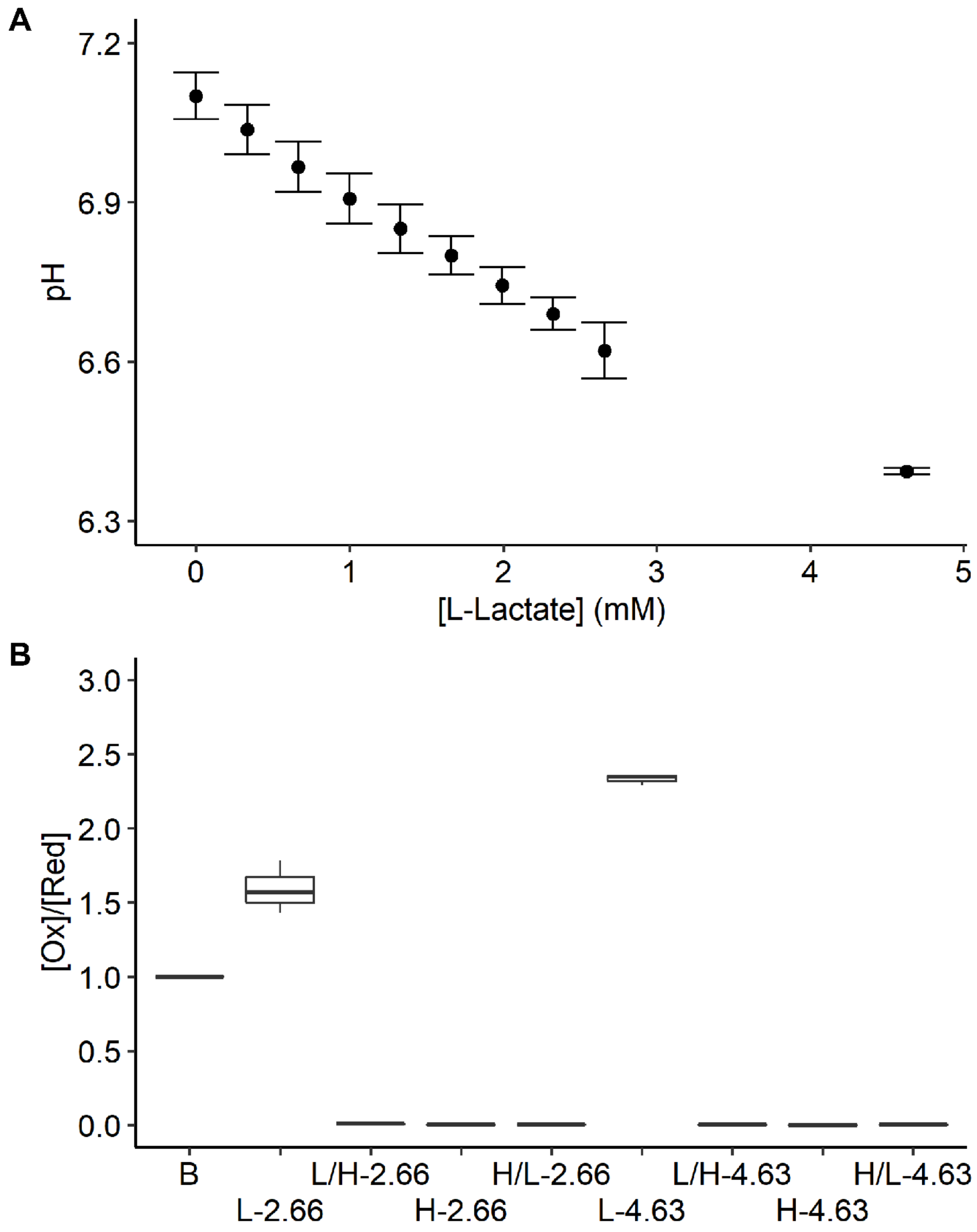

FIG S13 Determining the contribution of L-lactic acid and H_2O_2 to redox potential. **(A)** pH profile when L-lactate is added to TSB/CAA media. **(B)** [Ox]/[Red] ratios with various combinations of molecules: Media alone (B), 2.66 mM L-lactic acid (L-2.66), 2.66 mM L-lactic acid then H_2O_2 (L/H-2.66), 2.66 mM H_2O_2 (H-2.66), 2.66 mM H_2O_2 then L-lactic acid (H/L-2.66), 4.63 mM L-lactic acid (L-4.63), 4.63 mM L-lactic acid then H_2O_2 (L/H-4.63), 4.63 mM H_2O_2 (H-4.63), 4.63 mM H_2O_2 then L-lactic acid (H/L-4.63). All combinations of molecules were added in a 1:1 ratio (n=3 for all experiments).

TABLE S1 The drop method provides similar results to the conventional approach curve technique. Limiting current differences between electrodes measuring 1.5 mM FcMeOH in 1X PBS in the 20-30 µL drop as compared to completely submerged in solution. The limiting current differences are minimal between the drop and in solution for all UMEs. The average % current change for all five replicates was 2.88% ± 0.38%.

UME	25 µm Au	10 µm Pt	25 µm Pt	25 µm Pt	25 µm Pt
Drop	6.38 nA	1.89 nA	4.66 nA	4.90 nA	4.36 nA
Covered	6.42 nA	1.91 nA	4.73 nA	4.95 nA	4.82 nA
% Change	0.59%	1.18%	1.31%	0.90%	10.41%

TABLE S2 Expected vs. actual approach distances to disc surface. To calculate values, it was first estimated how far from the surface the UME approached using the first approach curve and then the electrode was retracted 100 µm. The system was then washed 3X with the same 1.5 mM FcMeOH in 1X PBS solution and the disc was submerged completely in the same solution. The electrode approached the disc surface again and the distance was measured from the surface (actual). The expected value was back calculated by adding the first approached value to the retracted value and then subtracting from the travel velocity multiplied by time travelled (expected).

UME	25 µm Au	10 µm Pt	25 µm Pt	25 µm Pt	25 µm Pt
Expected	15.83 µm	2.5 µm	18.03 µm	-1.03 µm	5.43 µm
	19.83 µm	3.85 µm	21.78 µm	3.23 µm	9.68 µm
Actual	25.13 µm	8.95 µm	21.13 µm	13.38 µm	14.38 µm
	33.13 µm	11.45 µm	27.13 µm	17.38 µm	18.63 µm

TABLE S3 Rates of H_2O_2 consumption determined using RespR. Maximum rates correspond to the most active region and were calculated at the beginning of the curve. Minimum Rates correspond to the most inactive region with the smallest decreasing slope. The best-fit rates correspond to the most linear region of each curve. See **Fig. 3** for visuals.

Condition	Max Rate	Min Rate	Best-Fit Rate
wt A. actinomycetemcomitans + H_2O_2	-0.107 mM/s	-0.015 mM/s	-0.032 mM/s
wt A. actinomycetemcomitans no H_2O_2	-0.009 mM/s	0.012 mM/s	-0.008 mM/s
A. actinomycetemcomitans katA$^-$ + H_2O_2	-0.045 mM/s	0.006 mM/s	-0.011 mM/s
A. actinomycetemcomitans katA$^-$ no H_2O_2	-0.009 mM/s	0.014 mM/s	0.005 mM/s
Media alone	-0.038 mM/s	0.011 mM/s	0.002 mM/s

TABLE S4 Maximum redox potentials and redox activity surrounding biofilms. Average redox potential (mV) is shown. These average redox activity values are graphically represented in **Fig. 4F** (n=3).

	**Average Maximum Redox Potential (mV) ± St. Dev.**	**Average Redox Activity ([Ox]/[Red]) ± St. Dev.**
wt A. actinomycetemcomitans	13.5 ± 4.28	1.68 ± 0.27
S. gordonii	23.0 ± 5.43	2.44 ± 0.51
wt A. actinomycetemcomitans/S. gordonii	22.9 ± 3.94	2.41 ± 0.38
P. aeruginosa	1.4 ± 0.81	1.06 ± 0.03
Media alone	-2.1×10^{-10} ± 0.00020	1.00 ± 0.007
